# Supplementary material for: Randomised controlled trial combining vitamin E-functionalised chocolate with physical exercise to reduce the risk of protein–energy malnutrition in predementia aged people: study protocol for Choko-Age
Source: BMJ Open. 2023 Dec 22;13(12):e072291. doi: 10.1136/bmjopen-2023-072291 (PMC10749016; doi:10.1136/bmjopen-2023-072291)
Supplement: Author's manuscript [file bmjopen-2023-072291.draft_revisions.pdf]

BMJ Open

BMJ Open is committed to open peer review. As part of this commitment we make the peer review history of every article we publish publicly available.

When an article is published we post the peer reviewers' comments and the authors' responses online. We also post the versions of the paper that were used during peer review. These are the versions that the peer review comments apply to.

The versions of the paper that follow are the versions that were submitted during the peer review process. They are not the versions of record or the final published versions. They should not be cited or distributed as the published version of this manuscript.

BMJ Open is an open access journal and the full, final, typeset and author-corrected version of record of the manuscript is available on our site with no access controls, subscription charges or pay-per-view fees (http://bmjopen.bmj.com).

If you have any questions on BMJ Open's open peer review process please email info.bmjopen@bmj.com

BMJ Open

Randomized Controlled Trial Combining vitamin E-functionalized Chocolate with physical exercise to reduce the risk of protein-energy malnutrition in pre-dementia Aged people: Study protocol for Choko-Age

Journal:	*BMJ Open*
Manuscript ID	bmjopen-2023-072291
Article Type:	Protocol
Date Submitted by the Author:	30-Jan-2023
Complete List of Authors:	Pedrinolla, Anna ; University of Trento, Cellular, Computational and Integrative Biology<br>Isanejad, Masoud; University of Liverpool, Musculoskeletal & Ageing Science<br>Antognelli, Cinzia ; Università degli Studi di Perugia, Department of Medicine and Surgery<br>Bartolini, Desirée; Università degli Studi di Perugia, Department of Medicine and Surgery, Bioscience and Medical Embryology Division<br>Borras, Consuelo ; University of Valencia, Department of Physiology, Faculty of Medicine<br>Cavedon, Valentina; University of Verona, of the Department of Neuroscience, Biomedicine and Movement (DNBM)<br>Di Sante, Gabriele ; University of Verona, Department of Neurosciences, biomedicine and Movement Sciences<br>Migni, Anna ; University of Perugia, Department of Pharmaceutical Sciences, Lipidomics and Micronutrient<br>Mas-Bargues, Cristina ; University of Valencia, Freshage Research Group, Department of Physiology, Faculty of Medicine, University of Valencia, Centro de Investigación Biomédica en Red Fragilidad y Envejecimiento Saludable-Instituto de Salud Carlos III (CIBERFES-ISCIII)<br>Milanese, Chiara ; University of Verona, Department of Neuroscience, Biomedicine and Movement Sciences<br>Baschirotto, Claudia ; University of Verona, Department of Neurosciences, biomedicine and Movement Sciences<br>Modena, Roberto ; Molde University College, Department of Health and Social Sciences<br>Pistilli , Alessandra ; University of Verona, of the Department of Neuroscience, Biomedicine and Movement<br>Rende , Mario ; University of Verona, of the Department of Neuroscience, Biomedicine and Movement (DNBM)<br>Schena, Federico; University of Verona, Department of Neuroscience, Biomedicine and Movement Sciences<br>Stabile , Anna Maria; University of Verona, of the Department of Neuroscience, Biomedicine and Movement (DNBM)<br>Vincenzo Telesa, Nicola; University of Perugia, Department of Medicine and Surgery<br>Tortorella, Sara ; Molecular Horizon srl

	Hemmings , Kay ; University of Liverpool, Musculoskeletal & Ageing Science<br>Vina, Jose; University of Valencia, Department of Physiology, Faculty of Medicine<br>Wang , Eivind ; Molde University College, Department of Health and Social Sciences; St Olavs Hospital Trondheim University Hospital<br>McArdle, Anne ; University of Liverpool Institute of Ageing and Chronic Disease, MRC-Versus Arthritis Centre for Integrated Research into Musculoskeletal Ageing (CIMA), Musculoskeletal & Ageing Science,<br>Jackson, Malcolm; University of Liverpool, MRC-Versus Arthritis Centre for Integrated Research into Musculoskeletal Ageing (CIMA), Musculoskeletal & Ageing Science<br>Venturelli , Massimo ; University of Verona, of the Department of Neuroscience, Biomedicine and Movement<br>Galli, Francesco; University of Perugia, Department of Pharmaceutical Sciences
Keywords:	Dementia, Musculoskeletal disorders < ORTHOPAEDIC & TRAUMA SURGERY, NUTRITION & DIETETICS

SCHOLARONE™
Manuscripts

Randomized Controlled Trial Combining vitamin E-functionalized Chocolate with physical exercise to reduce the risk of protein-energy malnutrition in pre-dementia Aged people: Study protocol for Choko-Age

Anna Pedrinolla[1*] & Masoud Isanejad[2*], Cinzia Antognelli[3], Desirée Bartolini[4], Consuelo Borras[5], Valentina Cavedon[6,] Gabriele Di Sante[7], Anna Migni[4], Cristina Mas-Bargues[5], Chiara Milanese[6], Claudia Baschirotto[6], Roberto Modena[8], Alessandra Pistilli [6], Mario Rende [6], Federico Schena [6], Anna Maria Stabile [6], Nicola Vincenzo Telesa [3], Sara Tortorella[9], Kay Hemmings [2] Jose Vina [5], Eivind Wang [8,10], Anne McArdle [2], Malcolm J Jackson[2], Massimo Venturelli [6], Francesco Galli [4] (the Choko-AgE consortium led by Galli Francesco).

[1]Cellular, Computational and Integrative Biology – CIBIO, University of Trento, Trento, Italy.

[2] MRC-Versus Arthritis Centre for Integrated Research into Musculoskeletal Ageing (CIMA), Department of Musculoskeletal and Ageing Science, Institute of Life Course and Medical Sciences, University of Liverpool, Liverpool L7 8TX, U.K.

[3] Department of Medicine and Surgery, Bioscience and Medical Embryology Division. P.le L. Severi 1 06132 Perugia, Italy

[4] Department of Pharmaceutical Sciences, Lipidomics and Micronutrient Vitamins Lab. and Human Anatomy Lab., University of Perugia, 06126 Perugia, Italy.

[5] Freshage Research Group, Department of Physiology, Faculty of Medicine, University of Valencia, Centro de Investigación Biomédica en Red Fragilidad y Envejecimiento Saludable-Instituto de Salud Carlos III (CIBERFES-ISCIII), INCLIVA, Valencia, 46010, Spain

[6] Department of Neurosciences, biomedicine and Movement Sciences, Univeristy of Verona, Verona, Italy

[7]Department of Medicine and Surgery, Section of Human, Clinical and Forensic Anatomy. P.le L. Severi 1 06132 Perugia

[8] Department of Health and Social Sciences, Molde University College, Norway

[9] Molecular Horizon s.r.l., Via Montelino 30, 06084 Bettona, Italy.

For peer review only

[10] Department of Østmarka, Division of Mental Health Care, St. Olavs Hospital, Trondheim University Hospital, Trondheim, Norway

Corresponding authors: Anna Pedrinolla (anna.pedrinolla@univr.it) and Masoud Isanejad (masoud.isanejad@liverpool.ac.uk) contributed equally to this work.

Keywords: Vitamin E, Polyphenols, Malnutrition, Exercise, Sarcopenia, Dementia

Word Count: 6265

Abstract

Objective: protein energy malnutrition (PEM) and the subsequent muscle wasting (sarcopenia) are common ageing complications. It is knowing to be also associated with dementia. Our programme will test the cytoprotective functions of vitamin E combined with the cortisol lowering effect of chocolate polyphenols, in combination with muscle anabolic effect of adequate dietary protein intake and physical exercise to prevent the age-dependent decline of muscle mass and its key underpinning mechanisms including mitochondrial function, and nutrient metabolism in muscle in the elderly.

Methods and analysis: in 2020, a 6-month double-blind randomized controlled trial in 75 pre-dementia older people was launched to prevent muscle mass loss, in respond to the 'Joint Programming Initiative A healthy diet for a healthy life'. In the run-in phase participants will be stabilized on protein rich diet (0.9-1.0 g protein/Kg ideal body weight/day) and physical exercise program (High Intensity Interval Training specifically developed for these subjects). Subsequently they will be randomized in to 3 groups (1:1:1). The study arms will have similar isocaloric diet and follow similar physical exercise programme. Control group (n=25) will maintain the baseline diet; intervention groups will consume either 30 g/day of dark chocolate containing 500 mg total polyphenols (corresponding to 60 mg epicatechin) and 100 mg vitamin E (as RRR-alpha-tocopherol) (n=25); or the high polyphenol chocolate without additional vitamin E (n=25). Muscle mass will be the primary endpoint. Other outcomes are neurocognitive status and previously identified biomolecular indices of frailty in pre-dementia patients. Muscle biopsies will be collected to assess myocyte contraction and mitochondrial metabolism. Blood and plasma samples will be analysed for laboratory endpoints including nutrition metabolism and omics.

For peer review only

Ethics and dissemination: issued by the ethical committees of the Azienda Ospedaliera Universitaria Integrata of Verona with respect to scientific content and compliance with applicable research and human subjects' regulation.

Trial registration number: NCT05343611, recruiting.

Strengths and limitations of this study

- This protocol paper presents a novel double-blinded intervention program that focuses on nutrition and exercise for older people with pre-dementia. The program is designed to improve protein energy malnutrition, muscle mass, functional and cognitive abilities.
- The study enables the acquisition of high-quality biological human data to inform the mechanism of muscle mass decline in older people.
- The study and its results are assumed to be applicable among older people as effective intervention strategy.
- Those unable to follow the exercise programme due to disability were not included to the study.

For peer review only

Introduction

Protein-energy malnutrition (PEM) is a condition caused by insufficient intake of protein and energy, and it is a significant concern for older adults. Current data shows that 23% of European older adults at *high* risk, and 48% at *some* risk of PEM (1). PEM is a significant risk factor for loss of muscle mass, physical function (i.e., sarcopenia), frailty (1–3) , and cognitive decline (4,5).

Cognitive decline and dementia are commonly linked to a decrease in muscle mass and physical function in older adults. This may be caused by an abnormal regulation of the hypothalamic-pituitary-adrenal (HPA) axis, leading to a disruption in the production of glucose ,reduction in energy supply to the muscles. (6). The , and chronically elevated cortisol level (7,8). Added to this, impaired energy and tissue anabolism pathways, cellular redox and ROS signaling are shown to accumulatively increase tissue degeneration in older people (9). Other possible mechanism of tissue damage are defective modulation of genes involved in immuno-inflammatory pathways causing low-grade inflammation (10), and hypercortisolism (8).

Combined nutrition and physical exercise interventions are considered a promising strategy to combat PEM, muscle mass and physical function loss in older age(3,5) but the optimal protocols required are yet to be determined. Dietary protein is a pre-requisite for the maintenance of skeletal muscle mass; stimulating muscle protein synthesis , and it has been shown to overcome anabolic resistance and muscle mass preservation in malnourished older individuals(12). Interestingly vitamin E supplementation improves myotube survival and prevents myoblasts from atrophy, also stimulating their proliferation and promoting membrane repair during exposure to oxidant challenges which attenuate senescence and replenish the regenerative capacity of these cells(13,14). Further, polyphenols (PP), are associated with inhibition of inflammatory cytokines, oxidative stress, and muscle atrophy-related ubiquitin ligases(15,16). Also, PP support activation of insulin growth factor-1 (IGF-1) signaling pathway and mitochondrial biogenesis and function, and differentiation factors involved in myogenesis(15).

A nutritional intervention to counteract PEM should preferably be combined with physical exercise. Previous randomized controlled trails (RCTs) shown the muscle anabolic exercise effect on muscle mass and strength (Wang et al. 2017) and on the

For peer review only - http://bmjopen.bmj.com/site/about/guidelines.xhtml

HPA axis reactivity and attenuation of hypercortisolism in patients with Alzheimer disease (17). Particularly, the maximal strength training (MST) has previously yielded excellent results in older subjects (18) . Also the high intensity interval training (HIIT), was previously shown to be excellent for improving health and performance in older subjects (19)and frail patient populations (20). These results sugegst that concurrent interventions consisting of both MST and HIIT can induce complementary, beneficial effects on muscle strength, mass, and metabolism (21).The effect of polyphenols or vitamin E combined with effective, HIIT, to improve muscle metabolism has not been investigated. Some animal studies have provided evidence that combination of exercise, PP, and vitamins (i.e., vitamin C and E) improved cellular antioxidant enzyme contents and reduced ROS levels(22,23). However, most of the findings obtained from *in vitro* studies and animal models have not been confirmed or fully investigated in clinical trials.

The hypothesis of the Choko-Age RCT is that the antioxidant and cytoprotective functions of vitamin E (24), combined with cortisol lowering effect of chocolate poly phenols (25), are augmented by HIIT and complemented with MST. In combination these may provide an optimal basis for an increase in muscle mass (primary end-point in the study) in elderly patients affected by cognitive decline. The Choko-Age RCT takes advantage of an innovative vitamin E-functionalized chocolate rich in poly phenols in combination with high intensity strength and endurance exercise to address PEM in community living older people with cognitive decline. The RCT protocol encompass a wide range of clinical and molecular investigations of skeletal muscle mitochondrial function, nutrient metabolism, insulin, and cortisol signaling, omics and tracing the enzymatic and free radical-derived metabolites of vitamin E for the first time in the human muscle.

For peer review only

Methods

Study design

This is a multicenter study, screening for eligible participants will be from 4 centers, Section of Movement Science of the Department of Neuroscience, Biomedicine, and Movement Science of University of Verona, Italy; Centro di Ricerca Sport Montagna e Salute (CeRiSM) – Rovereto, Italy; P. Pederzolli hospital – Peschiera, Italy; and Mons.

Mazzali Foundation – Mantua, Italy. The study has been registered with ClinicalTrials.gov Identifier: NCT05343611.

Eligibility criteria

The inclusion criteria are age 65-years and older; presence of mild cognitive impairment (MCI), subjective cognitive decline without functional deficit or mild dementia, in absence of severe chronic disease that compromises exercise and food metabolism. Mini Mental State (MMSE): cut-off 12 out of 30. The exclusion criteria are the presence of cancer and cancer-related cachexia, coagulation disorders, presence of diagnosed musculoskeletal diseases; inability to cooperate, regular users of any anticoagulant medication, or antiplatelet medications in high dose (es: acetylsalicylic acid >200mg per day).

Intervention description

After receiving consent, eligible participants will enter the 2-week *Run-in phase*, to stabilize the dietary protein intake at 0.9-1 g/ kg ideal body weight and inform participants about the physical exercise program. Diet quantity and quality will be assessed using a food frequency questionnaire by a dietitian and tailored dietary advice will be provided to maintain the required calorie and protein intake All participants will take part in the supervised physical exercise program consists of high intensity aerobic endurance exercise and HIIT three times a week, and each session will last about 50 minutes. The high intensity aerobic endurance training is carried out walking on a treadmill with 4 x 4 minutes at 85-95% of HRmax, interrupted by 3-minute active recovery periods (~60-70% of HRmax) (26). The high intensity strength training (maximal strength training; MST) is carried out in a seated horizontal leg press apparatus and performed as 4 sets with 4 repetitions at ~90% of maximal strength (1RM) (27). Rest periods between the sets will be 3–4 min. The training program will take place at each centre in thes multicentre study, where a room will be furnished with all the equipment needed. Physical exercise sessions will be supervised by a trained research team member and the ratio of supervisor: participants will be 1:2.

After the run-in phase, participants will be randomized into three intervention groups (25 participants in each group). Group A (i.e. control group) will have a high protein diet and take part in the physical exercise programme three times per week, for participants in this arm the diet plan will be adjusted to receive the same overall intake

For peer review only

of calories and macronutrients (3 g of proteins, 4 g of carbohydrates, 11 g of fat, 4 g of fibers) that the chocolate products will provide to groups B and C. Group B will receive 30g of 85% dark chocolate high in poly phenols) (poly phenols ≥ 500 mg of poly phenols with ≥ 60 mg of epicatechin) in addition to high protein diet and physical exercise. Group C will receive 30 grams of 85% dark PPs functionalized chocolate with 100 mg vitamin-E per day in addition to high protein diet and physical exercise. On the days of physical exercise for the Vitamin-E/ poly phenols groups B and C, chocolate will be consumed in the ninety minutes after the end of the training. The final functionalized chocolate has been produced with a collaborating partner, Nestlè Italiana SPA (owner of "Perugina" brand of pure chocolate products). Figure 1 presents the study timeline for each participant.

Outcomes

The primary outcome is change of lower limb muscle mass derived from body composition measurements, and specifically the fat-free soft tissue mass (gr). The Secondary outcomes are quadriceps volume and cross-sectional area measured by ultrasound (cm^3); lower limb muscle force generation derived from maximal voluntary contraction; cognitive function derived from the neuropsychological test's battery, vascular function derived from vascular assessments that include: FMD test and sPLM test. Metabolic function derived from walking energy cost ($j \cdot kg^{-1} \cdot m^{-1}$) and maximal oxygen consumption (ml/min/kg) as well as lab outcomes explained further in the protocol. Physical function derived from a 6-minute walking test (6MWT, m), Time-Up-and-Go (TUG, s) and 30 seconds Chair-stand (number of repetitions). We will also investigate mitochondrial respiration derived from the maximal respiratory capacity (ETS) and in vitro force generation of type 1 and 2 fibers assessed on muscle fibers obtained from the muscle biopsy. Circadian cortisol curve will be obtained from salivary cortisol collected during the day; in addition, blood hormones: IGF-1 (ELISA test) and the steroids estradiol, estrone, estriol, progesterone and testosterone (GC-MS analysis) will be measured.

Clinical and physiological assessments

All assessments are presented in schematic Figure 2 for everyone.

Body composition

For peer review only

Dual X-Ray absorptiometry (DXA) will be used for segmental and total body muscle mass and fat mass and bone mineral density (28). The Quadriceps volume and cross-sectional area (CSA) will be measured by the same ultrasound device throughout the whole study (29).

Physical function tests

6-minute walking test (6MWT), time-up and go (TUG), and 30 seconds chair-stand test. For measuring strength component, individuals will undergo maximal Voluntary Activation (MVC) and electrically evoked potential, to estimate the role of central fatigue; rate of force development (RFD) and peak force will be assessed to provide data on the relative neural and muscular contribution, respectively, to the muscle force development. To assess the energy cost of Walking (Cw) and maximal oxygen consumption. Individuals will perform a 3-speed walking test, on a treadmill.

Cognitive assessment

Patients will undergo a full neuropsychological battery of tests to explore verbal, visuospatial memory, working memory, attention, dual task, and executive functions. The global cognitive functioning will be assessed with MMSE. The Neuropsychiatric Inventory (NPI) will be used to evaluate the presence, frequency, and severity of behavioral disorders. The Geriatric Depression Scale will be used to assess the presence and the severity of depression.

Vascular function

Vascular function will be assessed by means of Flow-mediated dilation (FMD) and single Passive-leg movement (Trinity et al. 2020).

Saliva Cortisol

Cortisol will be collected using salivette with a cotton swab (Sarsted, AG & Co. KG, Nümbrecht, GERMANY) at the beginning and at the end of the three months treatment. Swab samples will be collected: (a) to measure acute response to a single exercise training session, saliva samples will be collected before starting the sessions and right at the end of one of it; (b) to measure the circadian curve, participants will be asked to collect saliva samples in a regular day (i.e. without exercise training) at four specific time points (7am, 11am, 3pm, and 7pm) (32,33).

For peer review only

Blood assays

Whole blood will be examined for glycosylation and transcriptomics. Plasma samples will be obtained for protein oxidation, lipid peroxidation, general hormones, ELISA kit assays. Furthermore, the analysis will include GC-MS and LC-MS/MS determinations of plasma levels of chocolate polyphenols, and vitamin E metabolites (34). Serum sample will be analyzed for untargeted l metabolomics, this latter is expected to provide a generic fingerprint of the response to the proposed nutritional intervention and to training activities. PBMCs will be used to extract proteins and mRNA for the analysis of genes involved in vitamin E metabolism (such as CYP4F2).

RNA extraction

Total RNA containing miRNA will be isolated by the mirVana miRNA Isolation Kit (Ambion, Austin, TX) according to the manufacturer's directions. RNA concentration and purity will be assessed with 260/280 ratio using a Genequant Pro Classic spectrophotometer (GE Healthcare, Spain) and RNA integrity by capillary electrophoresis using the RNA 6000 Nano Lab-on-a-Chip kit and the Bioanalyzer 2100 (Agilent Technologies, Santa Clara, CA).

Gene expression profiling

mRNA profiling will be performed using Clariom D-Human Array (Thermo Fisher Scientific). This array comprises >542 000 transcripts constituting over 134 000 gene-level probe sets. Microarray experiments were conducted according to the manufacturer's instructions (Thermo Fisher Scientific). Briefly, 150-ng total RNA will be labeled using GeneChip WT Plus Reagent Kit. The labeling reaction will be hybridized on the array using Hybridization Oven 645 at 45°C for 16 hours. The arrays are stained with Fluidics Station 450 using fluidics script FS450_0001 and then scanned on a GeneChip Scanner 3000 7G. GeneChip Command Console software supplied by Thermo Fisher Scientific will be used to perform gene expression analysis.

Data Analysis of Microarrays

Data (.CEL files) are analyzed and filtered using the Partek Genomic Suite 6.6 software (Partek Inc., St. Louis, MO). Input files are normalized with the RMA algorithm for gene array on core meta probesets or miRNAs. A 1-way analysis of variance (ANOVA) will be performed with the Partek Genomics Suite across all samples.

For peer review only

Statistically significant small noncoding RNAs and mRNAs between the different groups studied will be identified by using a model ANOVA of $p < .05$. The imported data will be analyzed by principal component analysis (PCA) to determine the significant sources of variability in the data. Differentially expressed genes will be imported into Pathway Studio.

Muscle Biopsy

A sample of skeletal muscle will be obtained with a 13-gauge tru-cut needle from the vastus lateralis muscle. This will be used for the following assays: (A) Assessment of muscle cytokine mRNA for a panel of pro-inflammatory cytokines examined by quantitative real-time PCR (qPCR). RNA will be isolated using standard Trizol® extraction method and purified using RNeasy clean-up kit; cDNA will be synthesised using iScript first strand kit from 1 µg of isolated RNA. (B) Muscle redox status will be undertaken by analysis of the reduced and oxidised glutathione contents of the biopsy together with analysis of the redox status of mitochondria through analysis of the proportion of peroxiredoxin 3 in the oxidised form, and cytosol by analysis of the proportion of peroxiredoxin 2 in the oxidised form (Pugh et al. 2021). (C) Muscle proteomics: A global label -free proteomic approach will be used at Ultimate 3000 RSLC nano system coupled to a QExactive mass spectrometer. Data analysis will be performed using Proteome Discover and Peaks7 software (35). (D) Determination of mRNA expression: RNA samples will be processed by following the specific platform protocols and the results will be bioinformatically analyzed. Expression analysis software and pipelines will be used to analyse the differential expression profiles of the selected genes. We will also analyse SUB-NETWORKS; this is to see the relationships that exist between the different transcripts to try and find common molecular-pathways. (E) Mitochondrial respiration derived from the maximal respiratory capacity (ETS) and in vitro force generation of types 1 and 2 fibers assessed on muscle fibers obtained from the muscle biopsy (36) (F) Targeted metabolomics of chocolate polyphenols and vitamin E by Gas Chromatography Mass Spec and Liquid Chromatography.

Provision of metabolomics

Targeted metabolomics of chocolate polyphenols and vitamin E by GC-MS and LC-MS/MS of patient plasma; as well as Inflammatory cytokines in blood and mRNA in

For peer review only

muscle, identification of relevant pathways from global transcriptomics and proteomics of blood and muscle, redox measurements and oxidative stress-related parameters of frailty.

Sample size

We aim to recruit n=75 older individuals (men and women) based on the eligibility criteria. The n=75 (25 for each group) sample size was based on alpha = 0.05 and power = 0.8. Considering the rate of drop out expected (20% ±2%) (37), n=96 individuals will be initially recruited. The main outcome is "muscle mass", and for all the groups treatment duration will be 3 months. In the target population the loss of muscle mass estimated to be 1.0-1.5% (± 0.5%) (38). In Group A, which includes people undergoing targeted exercise, the expected increase in muscle mass is 1.5% (±0.5%) (38). In groups B and C, the median expected increase at second follow-up is 4% (±0.5%), and 2% (±0.5%) at 3 months (39). Correlation between repeated measures is assumed to be 0.5, variance explained by the between-subjects effect 6.25 and error variance 65. All estimates were performed using Stata v.16.1 (StataCorp LP, College Station, TX, USA) by "power repeated" command.

Allocation and blinding

This is a randomized, double blinded, controlled trial. A blocked randomization approach will be applied. A statistician will create a randomization list including the groups (A, B or C) allocated to each subject, the blocks (after specifying the block size) will be randomly chosen to determine the assignment of all participants. Neither the participants nor research team will know the assignments of the intervention groups including type of chocolate (Vitamin-E and poly phenols, or poly phenols). Also, the participant in the control group (A) will not be aware of the chocolate consumption available for groups B and C.

Patient and Public Involvement

There were no implications for the patient and public involvement.

Statistical analysis

Statistical analysis will be conducted under the supervision of an expert in biostatistics and with the support of LIPOSTAR software. The primary data analysis will be two-way repeated measures ANOVA, including age and gender as covariates, with "time" as the within-group factor and "treatment" as the between-groups factor to calculate

For peer review only

difference between groups. In the presence of significant effects, a multiple comparisons tests with Bonferroni's correction will be performed. The familywise alpha level for significance will be set at 0.05 (two-tails), with Bonferroni's correction when needed, for all the analyses. This study is inherently generating extensive clinical and omics data which will be analyzed using robust and appropriate data analysis and with machine learning algorithms.

Data monitoring

An external supervisor will serve as external scientific supervisor of the clinical trial to monitor the distinct phases of clinical trial from organisation to implementation of activities, data gathering and evaluation/interpretation. The quality assurance standards of the University of Verona will be adopted to monitor the clinical trial. A delegate of this University will be nominated to perform the monitoring of the distinct phases of the trial utilising internal SOPs. The full set of clinical procedures, operator's activity and collection of experimental data will be verified during a series of visits by the monitor that will occur at the beginning and the end of each time point in the study (Time T00 to T2)

Ethics and disseminations

This protocol and the informed consent forms have been reviewed and approved by the applicable ethical committees of the *Azienda Ospedaliera Universitaria Integrata* of Verona with respect to scientific content and compliance with applicable research and human subjects' regulation. Thereby, this protocol is ethically conducted following the last revision of the Declaration of Helsinki as well as the Declaration of Oviedo. The protocol of the study is designed and will be conducted in order to adhere to Good Clinical Practice principles and procedures and in compliance with the Italian legislation, as described in the following documents and accepted by the investigators of the study with their signature: a) ICH Harmonized Tripartite Guidelines for Good Clinical Practice 1996; b) Directive 91/507/EEC, The Rules Governing Medicinal Products in the European Community; c) D. L.vo n.211 of 24th June 2003; d) D. L.vo n.200 6th November 2007; e) D.M. 21st December 2007. NIH Clinical trial identification ID and number: JPI- ERAHDL NCT05343611.

For peer review only

Consent, confidentiality, declaration of interests

A member of the research team will introduce the trial to individuals who show an interest in taking part in the study and their caregivers. Individuals will also receive information sheets. A member of the research team will discuss the trial with potential participants and their caregivers considering the information provided in the information sheets. Individuals and caregivers will then be able to have an informed discussion with the member of the research team who will obtain written informed consent from the persons willing to participate in the trial. All study-related information will be stored securely at the study site. All participant information will be stored in locked file cabinets in area with limited access. All laboratory specimens, reports, data collection, process and administrative forms will be identified by a coded ID number only to maintain participant confidentiality. All records that contain names or other personnel identifier will be stored separately from study records identified by code number. All local databases will be secured with password-protected access systems. Participants information will be stored for 10 years. Forms, lists, logbook, appointment books, and any other listing that link to participant ID numbers to other identifying information will be stored in a separate, locked file in an area with limited access.

Conflict of Interest

None declared.

Data access

Access to the database to start a data entry session is protected by a username and password. The following precautionary measures are taken to ensure data privacy and to prevent data manipulation and loss: i) Access to data is restricted to authorized members only. The authorized members are the researchers directly involved in the study who will collect, analyze, and interpret the data; ii) The network is protected by a firewall; iii) Internet connection is encrypted with a digital certificate (SSL technology); iv) The database is on a server, password protected, which is changed periodically; v) Access to the database is password protected and is accessible only to those responsible for the center; vi) Periodic backups are performed. vii) and places of conservation (e.g., The paper materials related to clinical evaluations will be stored in cabinets, whose keys will be in possession only of the persons authorized by the persons in charge of the study). Personal data of the subjects will be exclusively

For peer review only

accessible for investigators and collaborators/co-investigators, monitors and auditors of the Promoter, and/or for the competent authority , in agreement with what is included in the Informed consent. The Coordinator and the Investigators will inform the patients in a clear and thorough way about the modalities of personal data treatment before their participation to the experiment, as established by the applicable Privacy Laws.

Dissemination policy

A consortium agreement has been established for the dissemination policy. Each Unit is encouraged to publish in written form, oral presentation or making public in any other form (including electronic publication on the internet), hereinafter referred to as the “Publication”, the results of the Project, including but not limited to solely generated Foreground, after providing a copy of the draft Publication to the other Parties at least thirty calendar days prior to submission for publication or disclosure. The other parties may, by giving written notice to the Unit requesting the Publication (a “Confidentiality Notice"), require the publishing Unit to: (a) amend the Publication in order to remove any of its Background that is Confidential Information for the duration of the confidentiality obligation, or to remove any of its Foreground; or (b) amend the Publication if it can reasonably demonstrate that the manuscript compromises disproportionally its academic or legitimate interests. The other Unit shall give such Confidentiality Notice within thirty calendar days after receipt of the draft Publication. The Confidentiality Notice shall contain specific details to be amended and the Party seeking disclosure shall delete said specific details in as far as they comprise the other Units’s Confidential Information from the Publication or amend said specific details as not to Consortium Agreement " Choko-Age project” disproportionally harm the requesting Unit's academic or legitimate interests. If no objection is made within the time limit stated above, the Publication is permitted.

If an objection has been raised the involved Units shall discuss how to overcome the justified grounds for the objection on a timely basis (for example by amendment to the planned Publication before publication). The objecting Unit shall not unreasonably continue the opposition if appropriate actions are performed following the discussion. In any event, any delays of a Publication shall not exceed a period of more than 90 calendar days from the date of the notification from the Unit seeking disclosure. Contributors to the results obtained through work on the Project published shall be named and accredited in the Publication in accordance with academic custom. Other

For peer review only

aspects concerning result dissemination and the communications strategy of the project are presented in the "Communication and Dissemination plan" enclosed as Annex IV to the present Agreement.

Author contributions

AP and MI contributed equally to this paper. VJ, ZL, EW, AM, MJ, MV, AP, MI and FG are named investigators for this RCT. The Choko-Age group authors (CA, DB, CB, AM, GD, GC, GS, KH, AM, CB, GM, BCM, RM, MP, AP, MR, FS, BS, MAS, VNT, ST, JV, LZ, EV, AM, MJ, MV, FG, MI, and AP) have all contributed to the conception and design of the work, data acquisition, drafting and revising the study and are accountable for its intellectual content. This is the first version of the protocol submitted to BMJ Open.

Funding statement

To intervene with high-calory micronutrient-rich diet in elderly patients at risk of PEM could be problematic for different reasons including the difficulty to find palatable food items suitable to satisfy these nutritional criteria. The need for specific innovation in this area of food industry and human nutrition has recently been addressed in the call PREVNUT promoted by the "Joined Programming Initiatives-Healthy Diet for a Healthy Life" (JPI-HDHL) supported by the EU program Horizon 2020 under the umbrella of ERA-Net/ERA-HDHL and the international network of funding organizations reported in (https://www.healthydietforhealthylife.eu/index.php/call-activities/calls/98-calls-site-restyling/583-era-hdhl-2020). This project has received funding from MUR, Ministero dell'Università e della Ricerca (DD17080-24/11/2021) (Italy); Instituto de Salud Carlos III (ISCIII) (Ref. AC20/00026) (Spain); RCN, The Research Council of Norway (Norway) and UKRI, Biotechnology and Biological Sciences Research Council (BBSRC) (grant number BB/V019821/1) (UK), under the umbrella of the European Joint Programming Initiative "A Healthy Diet for a Healthy Life" (JPI HDHL) and of the ERA-NET Cofund ERA-HDHL (GA N° 696295 of the EU Horizon 2020 Research and Innovation Programme).

For peer review only

References

1. Leij-Halfwerk S, Verwijs MH, van Houdt S, Borkent JW, Guaitoli PR, Pelgrim T, et al. Prevalence of protein-energy malnutrition risk in European older adults in community, residential and hospital settings, according to 22 malnutrition screening tools validated for use in adults ≥65 years: A systematic review and meta-analysis. Vol. 126, Maturitas. Elsevier Ireland Ltd; 2019. p. 80–9.

2. Eglseer D, Eminovic S, Lohrmann C. Association between sarcopenia and nutritional status in older adults: A systematic literature review. Vol. 42, Journal of Gerontological Nursing. Slack Incorporated; 2016. p. 33–41.

3. Mathewson SL, Azevedo PS, Gordon AL, Phillips BE, Greig CA. Overcoming protein-energy malnutrition in older adults in the residential care setting: A narrative review of causes and interventions. Ageing Res Rev [Internet]. 2021;70(May):101401. Available from: https://doi.org/10.1016/j.arr.2021.101401

4. Anderson R, Weindruch R. Metabolic reprogramming in dietary restriction. Vol. 35, Interdisciplinary Topics in Gerontology. Interdiscip Top Gerontol; 2007. p. 18–38.

5. Droogsma E, van Asselt D, de Deyn PP. Gewichtsverlust und Unterernährung bei selbstständig lebenden Patienten mit Alzheimer-Demenz: Von populationsbasierten Studien zum klinischen Management. Z Gerontol Geriatr. 2015;48(4):318–24.

6. Martocchia A, Gallucci M, Noale M, Maggi S, Cassol M, Stefanelli M, et al. The increased cortisol levels with preserved rhythmicity in aging and its relationship with dementia and metabolic syndrome. Aging Clin Exp Res. 2022;

7. Venturelli M, Cè E, Limonta E, Muti E, Scarsini R, Brasioli A, et al. Possible Predictors of Involuntary Weight Loss in Patients with Alzheimer's Disease. PLoS One. 2016;

8. Ouanes S, Popp J. High cortisol and the risk of dementia and alzheimer's disease: A review of the literature [Internet]. Vol. 11, Frontiers in Aging Neuroscience. Frontiers Media S.A.; 2019 [cited 2022 Jun 15]. Available from: https://pubmed.ncbi.nlm.nih.gov/30881301/

9. Oudbier SJ, Goh J, Looijaard SMLM, Reijnierse EM, Meskers CGM, Maier AB. Pathophysiological Mechanisms Explaining the Association Between Low Skeletal Muscle Mass and Cognitive Function. The Journals of Gerontology: Series A [Internet]. 2022 Oct 6 [cited 2022 Nov 5];77(10). Available from: https://pubmed.ncbi.nlm.nih.gov/35661882/

10. Beura SK, Dhapola R, Panigrahi AR, Yadav P, Reddy DH, Singh SK. Redefining oxidative stress in Alzheimer's disease: Targeting platelet reactive oxygen species for novel therapeutic options. Vol. 306, Life Sciences. Elsevier Inc.; 2022.

11. Isanejad M, Mursu J, Sirola J, Kröger H, Rikkonen T, Tuppurainen M, et al. Dietary protein intake is associated with better physical function and muscle strength among elderly women. British Journal of Nutrition. 2016 Feb 11;115(7):1281–91.

12. Strasser B, Wolters M, Weyh C, Krüger K, Ticinesi A. The effects of lifestyle and diet on gut microbiota composition, inflammation and muscle performance in our aging society. Nutrients. 2021;13(6).

13. Chung E, Mo H, Wang S, Zu Y, Elfakhani M, Rios SR, et al. Potential roles of vitamin E in age-related changes in skeletal muscle health. Nutrition Research. 2018;49:23–36.

14. Khor SC, Abdul Karim N, Wan Ngah WZ, Mohd Yusof YA, Makpol S. Vitamin e in sarcopenia: Current evidences on its role in prevention and treatment. Oxid Med Cell Longev. 2014;2014.

15. Nikawa T, Ulla A, Sakakibara I. Polyphenols and their effects on muscle atrophy and muscle health. Molecules. 2021;26(16).

16. Thoma A, Akter-Miah T, Reade RL, Lightfoot AP. Targeting reactive oxygen species (ROS) to combat the age-related loss of muscle mass and function. Biogerontology. 2020;21(4):475–84.

17. Pedrinolla A, Schena F, Venturelli M. Resilience to Alzheimer ' s Disease : The Role of Physical Activity. 2017;546–53.

For peer review only

18. Torvild Kittilsen H, Ingegerdsson Freberg B, Nicolaisen I, Maria Støa E, Bratland-Sanda S, Helgerud J, et al. Responses to Maximal Strength Training in Different Age and Gender Groups. Front Physiol [Internet]. 2021;12:636972. Available from: www.frontiersin.org

19. StØren Ø, Helgerud J, SÆbØ M, StØa EM, Bratland-Sanda S, Unhjem RJ, et al. The Effect of Age on the V-O2max Response to High-Intensity Interval Training. Med Sci Sports Exerc. 2017 Jan 1;49(1):78–85.

20. Helgerud J, Wang E, Mats ·, Mosti P, Øystein ·, Wiggen N, et al. Plantar Xexion training primes peripheral arterial disease patients for improvements in cardiac function. Eur J Appl Physiol. 2009;106:207–15.

21. Mosti MP, Wang E, Wiggen ØN, Helgerud J, Hoff J. Concurrent strength and endurance training improves physical capacity in patients with peripheral arterial disease. Scand J Med Sci Sports [Internet]. 2011;21:e308–14. Available from: https://onlinelibrary.wiley.com/doi/10.1111/j.1600-0838.2011.01294.x

22. Liu S, Meng F, Zhang D, Shi D, Zhou J, Guo S, et al. Lonicera caerulea Berry Polyphenols Extract Alleviates Exercise Fatigue in Mice by Reducing Oxidative Stress, Inflammation, Skeletal Muscle Cell Apoptosis, and by Increasing Cell Proliferation. Front Nutr. 2022;9:853225.

23. Mock JT, Chaudhari K, Sidhu A, Sumien N. The influence of vitamins E and C and exercise on brain aging. Exp Gerontol. 2017;94:69–72.

24. Galli F, Azzi A, Birringer M, Cook-Mills JM, Eggersdorfer M, Frank J, et al. Vitamin E: Emerging aspects and new directions. Vol. 102, Free Radical Biology and Medicine. Elsevier Inc.; 2017. p. 16–36.

25. Ammar A, Mounaturki, Trabelsi K, Bragazzi NL, Boukhris O, Bouaziz M, et al. Effects of natural polyphenol-rich pomegranate juice on the acute and delayed response of Homocysteine and steroidal hormones following weightlifting exercises: A double-blind, placebo-controlled trial. J Int Soc Sports Nutr. 2020 Mar 6;17(1).

For peer review only

26. Brobakken MF, Nyg ard M, Morken G, Reitan SK, Heggelund J, Wang E, et al. Aerobic interval training in standard treatment of out-patients with schizophrenia: a randomized controlled trial. Acta Psychiatr Scand [Internet]. 2019;140:498–507. Available from: https://onlinelibrary.wiley.com/doi/10.1111/acps.13105

27. Tøien T, Unhjem R, Øren TS, Kvellestad ACG, Hoff J, Wang E. Neural Plasticity with Age: Unilateral Maximal Strength Training Augments Efferent Neural Drive to the Contralateral Limb in Older Adults. Journals of Gerontology - Series A Biological Sciences and Medical Sciences. 2018;73(5):596–602.

28. Hart NH, Nimphius S, Spiteri T, Cochrane JL, Newton RU. Segmental musculoskeletal examinations using dual-energy X-ray absorptiometry (DXA): Positioning and analysis considerations. J Sports Sci Med. 2015 Sep 1;14(3):620–6.

29. Franchi M V., Fitze DP, Hanimann J, Sarto F, Spörri J. Panoramic ultrasound vs. MRI for the assessment of hamstrings cross-sectional area and volume in a large athletic cohort. Sci Rep. 2020 Dec 1;10(1).

30. Rossman MJ, Groot HJ, Garten RS, Witman MAH, Richardson RS. Vascular function assessed by passive leg movement and flow-mediated dilation: initial evidence of construct validity. American Journal of Physiology-Heart and Circulatory Physiology. 2016;311(5):H1277–86.

31. Trinity JD, Kwon OS, Broxterman RM, Gifford JR, Kithas AC, Hydren JR, et al. The role of the endothelium in the hyperemic response to passive leg movement: looking beyond nitric oxide. American Journal of Physiology-Heart and Circulatory Physiology. 2020 Dec 11;ajpheart.00784.2020.

32. Venturelli M, Amann M, Layec G, Mcdaniel J, Trinity JD, Fjeldstad AS, et al. Passive leg movement-induced hyperaemia with a spinal cord lesion: Evidence of preserved vascular function. Acta Physiologica. 2014 Feb;210(2):429–39.

33. Pedrinolla A, Tamburin S, Brasioli A, Sollima A, Fonte C, Muti E, et al. An Indoor Therapeutic Garden for Behavioral Symptoms in Alzheimer's Disease: A Randomized Controlled Trial. Journal of Alzheimer's Disease. 2019;71(3).

For peer review only

34. Bartolini D, Marinelli R, Giusepponi D, Galarini R, Barola C, Stabile AM, et al. Alpha-tocopherol metabolites (the vitamin E metabolome) and their interindividual variability during supplementation. Antioxidants. 2021 Feb 1;10(2):1–16.

35. Pugh JN, Stretton C, McDonagh B, Brownridge P, McArdle A, Jackson MJ, et al. Exercise stress leads to an acute loss of mitochondrial proteins and disruption of redox control in skeletal muscle of older subjects: An underlying decrease in resilience with aging? Free Radic Biol Med. 2021 Dec 1;177:88–99.

36. Claflin DR, Larkin LM, Cederna PS, Horowitz JF, Alexander NB, Cole NM, et al. Effects of high-and low-velocity resistance training on the contractile properties of skeletal muscle fibers from young and older humans. J Appl Physiol [Internet]. 2011;111:1021–30. Available from: http://www.jap.org

37. Pedrinolla A, Venturelli M, Fonte C, Munari D, Benetti MV, Rudi D, et al. Exercise training on locomotion in patients with Alzheimer's disease: A feasibility study. Journal of Alzheimer's Disease. 2018;61(4).

38. Vikberg S, Sörlén N, Brandén L, Johansson J, Nordström A, Hult A, et al. Effects of Resistance Training on Functional Strength and Muscle Mass in 70-Year-Old Individuals With Pre-sarcopenia: A Randomized Controlled Trial. J Am Med Dir Assoc. 2019;20(1):28–34.

39. Distefano G, Goodpaster BH. Effects of exercise and aging on skeletal muscle. Cold Spring Harb Perspect Med. 2018;8(3).

For peer review only

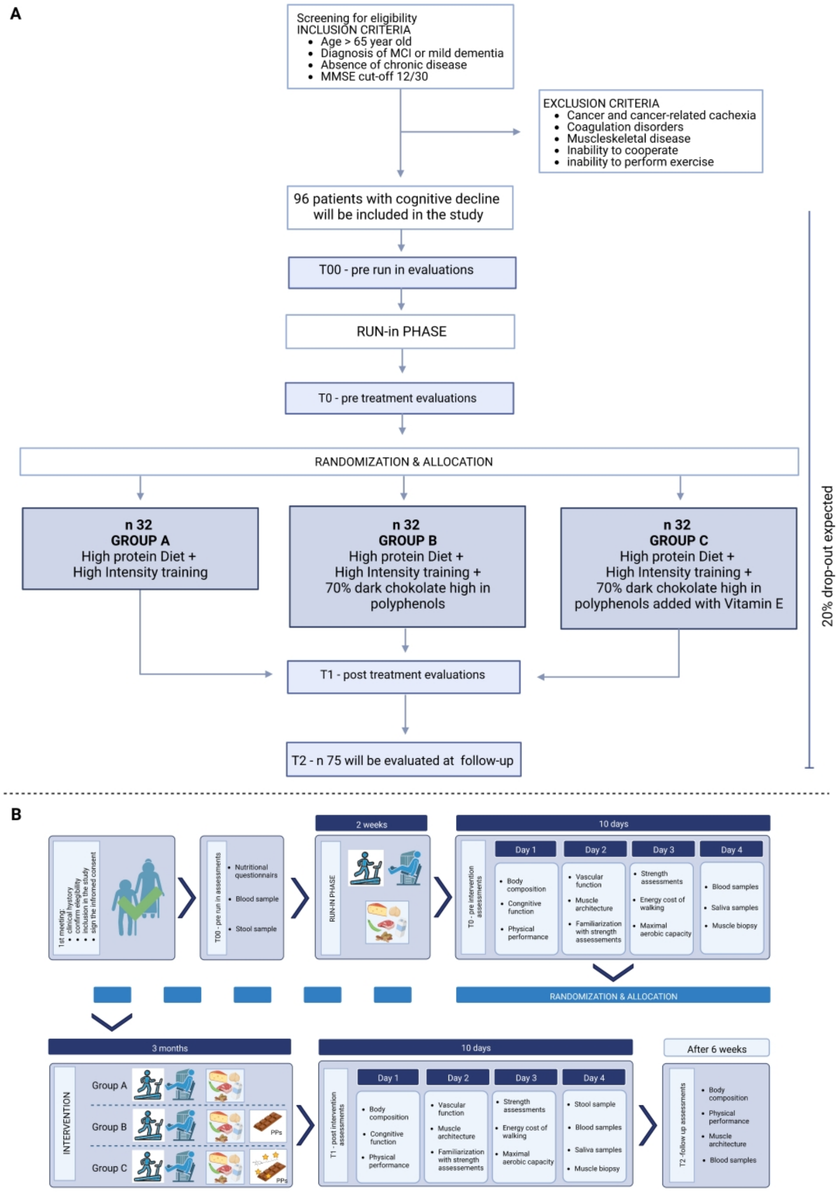

Individual timeline

305x425mm (300 x 300 DPI)

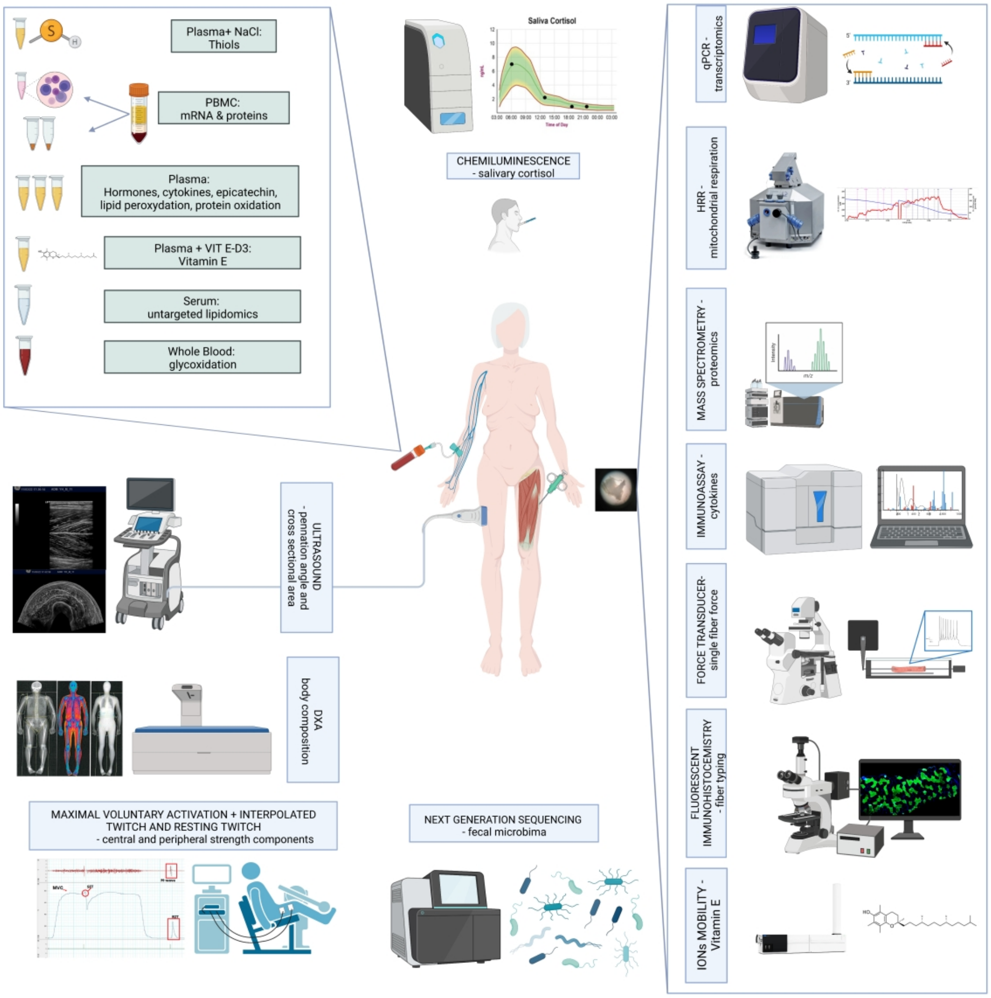

Study graphical abstract for clinical and laboratory endpoints

406x406mm (300 x 300 DPI)

Reporting checklist for protocol of a clinical trial.

Based on the SPIRIT guidelines.

Instructions to authors

Complete this checklist by entering the page numbers from your manuscript where readers will find each of the items listed below.

Your article may not currently address all the items on the checklist. Please modify your text to include the missing information. If you are certain that an item does not apply, please write "n/a" and provide a short explanation.

Upload your completed checklist as an extra file when you submit to a journal.

In your methods section, say that you used the SPIRITreporting guidelines, and cite them as:

Chan A-W, Tetzlaff JM, Gøtzsche PC, Altman DG, Mann H, Berlin J, Dickersin K, Hróbjartsson A, Schulz KF, Parulekar WR, Krleža-Jerić K, Laupacis A, Moher D. SPIRIT 2013 Explanation and Elaboration: Guidance for protocols of clinical trials. BMJ. 2013;346:e7586

		Reporting Item	Page Number
**Administrative information**			
Title	#1	Descriptive title identifying the study design, population, interventions, and, if applicable, trial acronym	1
Trial registration	#2a	Trial identifier and registry name. If not yet registered, name of intended registry	4
Trial registration: data set	#2b	All items from the World Health Organization Trial Registration Data Set	9
Protocol version	#3	Date and version identifier	11
Funding	#4	Sources and types of financial, material, and other support	11
Roles and responsibilities: contributorship	#5a	Names, affiliations, and roles of protocol contributors	10

For peer review only - http://bmjopen.bmj.com/site/about/guidelines.xhtml

Roles and responsibilities: sponsor contact information	#5b	Name and contact information for the trial sponsor	NA
Roles and responsibilities: sponsor and funder	#5c	Role of study sponsor and funders, if any, in study design; collection, management, analysis, and interpretation of data; writing of the report; and the decision to submit the report for publication, including whether they will have ultimate authority over any of these activities	11
Roles and responsibilities: committees	#5d	Composition, roles, and responsibilities of the coordinating centre, steering committee, endpoint adjudication committee, data management team, and other individuals or groups overseeing the trial, if applicable (see Item 21a for data monitoring committee)	9
**Introduction**			
Background and rationale	#6a	Description of research question and justification for undertaking the trial, including summary of relevant studies (published and unpublished) examining benefits and harms for each intervention	1
Background and rationale: choice of comparators	#6b	Explanation for choice of comparators	1
Objectives	#7	Specific objectives or hypotheses	2
Trial design	#8	Description of trial design including type of trial (eg, parallel group, crossover, factorial, single group), allocation ratio, and framework (eg, superiority, equivalence, non-inferiority, exploratory)	3
**Methods: Participants, interventions, and outcomes**			
Study setting	#9	Description of study settings (eg, community clinic, academic hospital) and list of countries where data will be collected. Reference to where list of study sites can be	3

For peer review only

		obtained	
Eligibility criteria	#10	Inclusion and exclusion criteria for participants. If applicable, eligibility criteria for study centres and individuals who will perform the interventions (eg, surgeons, psychotherapists)	4
Interventions: description	#11a	Interventions for each group with sufficient detail to allow replication, including how and when they will be administered	4
Interventions: modifications	#11b	Criteria for discontinuing or modifying allocated interventions for a given trial participant (eg, drug dose change in response to harms, participant request, or improving / worsening disease)	4
Interventions: adherance	#11c	Strategies to improve adherence to intervention protocols, and any procedures for monitoring adherence (eg, drug tablet return; laboratory tests)	5
Interventions: concomitant care	#11d	Relevant concomitant care and interventions that are permitted or prohibited during the trial	4
Outcomes	#12	Primary, secondary, and other outcomes, including the specific measurement variable (eg, systolic blood pressure), analysis metric (eg, change from baseline, final value, time to event), method of aggregation (eg, median, proportion), and time point for each outcome. Explanation of the clinical relevance of chosen efficacy and harm outcomes is strongly recommended	5.6
Participant timeline	#13	Time schedule of enrolment, interventions (including any run-ins and washouts), assessments, and visits for participants. A schematic diagram is highly recommended (see Figure)	5
Sample size	#14	Estimated number of participants needed to achieve study objectives and how it was determined, including clinical and statistical assumptions supporting any sample size calculations	8
Recruitment	#15	Strategies for achieving adequate participant enrolment to reach target sample size	8

For peer review only

**Methods: Assignment of interventions (for controlled trials)**			
Allocation: sequence generation	#16a	Method of generating the allocation sequence (eg, computer-generated random numbers), and list of any factors for stratification. To reduce predictability of a random sequence, details of any planned restriction (eg, blocking) should be provided in a separate document that is unavailable to those who enrol participants or assign interventions	8
Allocation concealment mechanism	#16b	Mechanism of implementing the allocation sequence (eg, central telephone; sequentially numbered, opaque, sealed envelopes), describing any steps to conceal the sequence until interventions are assigned	8
Allocation: implementation	#16c	Who will generate the allocation sequence, who will enrol participants, and who will assign participants to interventions	8
Blinding (masking)	#17a	Who will be blinded after assignment to interventions (eg, trial participants, care providers, outcome assessors, data analysts), and how	8
Blinding (masking): emergency unblinding	#17b	If blinded, circumstances under which unblinding is permissible, and procedure for revealing a participant's allocated intervention during the trial	8
**Methods: Data collection, management, and analysis**			
Data collection plan	#18a	Plans for assessment and collection of outcome, baseline, and other trial data, including any related processes to promote data quality (eg, duplicate measurements, training of assessors) and a description of study instruments (eg, questionnaires, laboratory tests) along with their reliability and validity, if known. Reference to where data collection forms can be found, if not in the	9

For peer review only

		protocol	
Data collection plan: retention	#18b	Plans to promote participant retention and complete follow-up, including list of any outcome data to be collected for participants who discontinue or deviate from intervention protocols	9
Data management	#19	Plans for data entry, coding, security, and storage, including any related processes to promote data quality (eg, double data entry; range checks for data values). Reference to where details of data management procedures can be found, if not in the protocol	9
Statistics: outcomes	#20a	Statistical methods for analysing primary and secondary outcomes. Reference to where other details of the statistical analysis plan can be found, if not in the protocol	5
Statistics: additional analyses	#20b	Methods for any additional analyses (eg, subgroup and adjusted analyses)	8
Statistics: analysis population and missing data	#20c	Definition of analysis population relating to protocol non-adherence (eg, as randomised analysis), and any statistical methods to handle missing data (eg, multiple imputation)	8
**Methods: Monitoring**			
Data monitoring: formal committee	#21a	Composition of data monitoring committee (DMC); summary of its role and reporting structure; statement of whether it is independent from the sponsor and competing interests; and reference to where further details about its charter can be found, if not in the protocol. Alternatively, an explanation of why a DMC is not needed	8
Data monitoring: interim analysis	#21b	Description of any interim analyses and stopping guidelines, including who will have access to these interim results and make the final decision to terminate the trial	8
Harms	#22	Plans for collecting, assessing, reporting, and managing solicited and spontaneously reported adverse events and other unintended effects of trial interventions or trial conduct	9
Auditing	#23	Frequency and procedures for auditing trial conduct, if	9

For peer review only

		any, and whether the process will be independent from investigators and the sponsor	
**Ethics and dissemination**			
Research ethics approval	#24	Plans for seeking research ethics committee / institutional review board (REC / IRB) approval	9
Protocol amendments	#25	Plans for communicating important protocol modifications (eg, changes to eligibility criteria, outcomes, analyses) to relevant parties (eg, investigators, REC / IRBs, trial participants, trial registries, journals, regulators)	11
Consent or assent	#26a	Who will obtain informed consent or assent from potential trial participants or authorised surrogates, and how (see Item 32)	9
Consent or assent: ancillary studies	#26b	Additional consent provisions for collection and use of participant data and biological specimens in ancillary studies, if applicable	9
Confidentiality	#27	How personal information about potential and enrolled participants will be collected, shared, and maintained in order to protect confidentiality before, during, and after the trial	9
Declaration of interests	#28	Financial and other competing interests for principal investigators for the overall trial and each study site	9
Data access	#29	Statement of who will have access to the final trial dataset, and disclosure of contractual agreements that limit such access for investigators	9
Ancillary and post trial care	#30	Provisions, if any, for ancillary and post-trial care, and for compensation to those who suffer harm from trial participation	NA
Dissemination policy: trial results	#31a	Plans for investigators and sponsor to communicate trial results to participants, healthcare professionals, the public, and other relevant groups (eg, via publication, reporting in results databases, or other data sharing arrangements), including any publication restrictions	10
Dissemination policy:	#31b	Authorship eligibility guidelines and any intended use of	10

For peer review only

authorship		professional writers	
Dissemination policy: reproducible research	#31c	Plans, if any, for granting public access to the full protocol, participant-level dataset, and statistical code	10

Appendices

Informed consent materials	#32	Model consent form and other related documentation given to participants and authorised surrogates	NA
Biological specimens	#33	Plans for collection, laboratory evaluation, and storage of biological specimens for genetic or molecular analysis in the current trial and for future use in ancillary studies, if applicable	NA

The SPIRIT Explanation and Elaboration paper is distributed under the terms of the Creative Commons Attribution License CC-BY-NC. This checklist was completed on 26. January 2023 using https://www.goodreports.org/, a tool made by the EQUATOR Network in collaboration with Penelope.ai

For peer review only

BMJ Open

Randomized Controlled Trial Combining vitamin E-functionalized Chocolate with physical exercise to reduce the risk of protein-energy malnutrition in pre-dementia Aged people: Study protocol for Choko-Age

Journal:	*BMJ Open*
Manuscript ID	bmjopen-2023-072291.R1
Article Type:	Protocol
Date Submitted by the Author:	31-Aug-2023
Complete List of Authors:	Pedrinolla, Anna ; University of Trento, Cellular, Computational and Integrative Biology<br>Isanejad, Masoud; University of Liverpool, Musculoskeletal & Ageing Science<br>Antognelli, Cinzia ; Università degli Studi di Perugia, Department of Medicine and Surgery<br>Bartolini, Desirée; Università degli Studi di Perugia, Department of Medicine and Surgery, Bioscience and Medical Embryology Division<br>Borras, Consuelo ; University of Valencia, Department of Physiology, Faculty of Medicine<br>Cavedon, Valentina; University of Verona, of the Department of Neuroscience, Biomedicine and Movement (DNBM)<br>Di Sante, Gabriele ; University of Verona, Department of Neurosciences, biomedicine and Movement Sciences<br>Migni, Anna ; University of Perugia, Department of Pharmaceutical Sciences, Lipidomics and Micronutrient<br>Mas-Bargues, Cristina ; University of Valencia, Freshage Research Group, Department of Physiology, Faculty of Medicine, University of Valencia, Centro de Investigación Biomédica en Red Fragilidad y Envejecimiento Saludable-Instituto de Salud Carlos III (CIBERFES-ISCIII)<br>Milanese, Chiara ; University of Verona, Department of Neuroscience, Biomedicine and Movement Sciences<br>Baschirotto, Claudia ; University of Verona, Department of Neurosciences, biomedicine and Movement Sciences<br>Modena, Roberto ; Molde University College, Department of Health and Social Sciences<br>Pistilli , Alessandra ; University of Verona, of the Department of Neuroscience, Biomedicine and Movement<br>Rende , Mario ; University of Verona, of the Department of Neuroscience, Biomedicine and Movement (DNBM)<br>Schena, Federico; University of Verona, Department of Neuroscience, Biomedicine and Movement Sciences<br>Stabile , Anna Maria; University of Verona, of the Department of Neuroscience, Biomedicine and Movement (DNBM)<br>Vincenzo Telesa, Nicola; University of Perugia, Department of Medicine and Surgery<br>Tortorella, Sara ; Molecular Horizon srl

	Hemmings , Kay ; University of Liverpool, Musculoskeletal & Ageing Science<br>Vina, Jose; University of Valencia, Department of Physiology, Faculty of Medicine<br>Wang , Eivind ; Molde University College, Department of Health and Social Sciences; St Olavs Hospital Trondheim University Hospital<br>McArdle, Anne ; University of Liverpool Institute of Ageing and Chronic Disease, MRC-Versus Arthritis Centre for Integrated Research into Musculoskeletal Ageing (CIMA), Musculoskeletal & Ageing Science,<br>Jackson, Malcolm; University of Liverpool, MRC-Versus Arthritis Centre for Integrated Research into Musculoskeletal Ageing (CIMA), Musculoskeletal & Ageing Science<br>Venturelli , Massimo ; University of Verona, of the Department of Neuroscience, Biomedicine and Movement<br>Galli, Francesco; University of Perugia, Department of Pharmaceutical Sciences
**Primary Subject Heading**:	Nutrition and metabolism
Secondary Subject Heading:	Complementary medicine
Keywords:	Dementia, Musculoskeletal disorders < ORTHOPAEDIC & TRAUMA SURGERY, NUTRITION & DIETETICS

SCHOLARONE™ Manuscripts

Randomized Controlled Trial Combining Vitamin E-functionalized Chocolate with physical exercise to reduce the risk of protein-energy malnutrition in pre-dementia Aged people: Study protocol for Choko-Age

Anna Pedrinolla[1*] & Masoud Isanejad[2*], Cinzia Antognelli[3], Desirée Bartolini[4], Consuelo Borras[5], Valentina Cavedon[6,] Gabriele Di Sante[7], Anna Migni[4], Cristina Mas-Bargues[5], Chiara Milanese[6], Claudia Baschirotto[6], Roberto Modena[8], Alessandra Pistilli [6], Mario Rende [6], Federico Schena [6], Anna Maria Stabile [6], Nicola Vincenzo Telesa [3], Sara Tortorella[9], Kay Hemmings [2] Jose Vina [5], Eivind Wang [8,10], Anne McArdle [2], Malcolm J Jackson[2], Massimo Venturelli [6], Francesco Galli

[1]Cellular, Computational and Integrative Biology – CIBIO, University of Trento, Trento, Italy.

[2] MRC-Versus Arthritis Centre for Integrated Research into Musculoskeletal Ageing (CIMA), Department of Musculoskeletal and Ageing Science, Institute of Life Course and Medical Sciences, University of Liverpool, Liverpool L7 8TX, U.K.

[3] Department of Medicine and Surgery, Bioscience and Medical Embryology Division. P.le L. Severi 1 06132 Perugia, Italy

[4] Department of Pharmaceutical Sciences, Lipidomics and Micronutrient Vitamins Lab. and Human Anatomy Lab., University of Perugia, 06126 Perugia, Italy.

[5] Freshage Research Group, Department of Physiology, Faculty of Medicine, University of Valencia, Centro de Investigación Biomédica en Red Fragilidad y Envejecimiento Saludable-Instituto de Salud Carlos III (CIBERFES-ISCIII), INCLIVA, Valencia, 46010, Spain

[6] Department of Neurosciences, biomedicine and Movement Sciences, Univeristy of Verona, Verona, Italy

[7]Department of Medicine and Surgery, Section of Human, Clinical and Forensic Anatomy. P.le L. Severi 1 06132 Perugia

[8] Department of Health and Social Sciences, Molde University College, Norway

[9] Molecular Horizon s.r.l., Via Montelino 30, 06084 Bettona, Italy.

For peer review only

[10] Department of Østmarka, Division of Mental Health Care, St. Olavs Hospital, Trondheim University Hospital, Trondheim, Norway

Corresponding authors: Anna Pedrinolla (anna.pedrinolla@univr.it) and Masoud Isanejad (masoud.isanejad@liverpool.ac.uk) contributed equally to this work.

Keywords: Vitamin E, Polyphenols, Malnutrition, Exercise, Sarcopenia, Dementia

Word Count: 6265

Abstract

Objective: protein energy malnutrition (PEM) and the subsequent muscle wasting (sarcopenia) are common ageing complications. It is knowing to be also associated with dementia. Our programme will test the cytoprotective functions of vitamin E combined with the cortisol lowering effect of chocolate polyphenols, in combination with muscle anabolic effect of adequate dietary protein intake and physical exercise to prevent the age-dependent decline of muscle mass and its key underpinning mechanisms including mitochondrial function, and nutrient metabolism in muscle in the elderly.

Methods and analysis: in 2020, a 6-month double-blind randomized controlled trial in 75 pre-dementia older people was launched to prevent muscle mass loss, in respond to the 'Joint Programming Initiative A healthy diet for a healthy life'. In the run-in phase participants will be stabilized on protein rich diet (0.9-1.0 g protein/Kg ideal body weight/day) and physical exercise program (High Intensity Interval Training specifically developed for these subjects). Subsequently they will be randomized in to 3 groups (1:1:1). The study arms will have similar isocaloric diet and follow similar physical exercise programme. Control group (n=25) will maintain the baseline diet; intervention groups will consume either 30 g/day of dark chocolate containing 500 mg total polyphenols (corresponding to 60 mg epicatechin) and 100 mg vitamin E (as RRR-alpha-tocopherol) (n=25); or the high polyphenol chocolate without additional vitamin E (n=25). Muscle mass will be the primary endpoint. Other outcomes are neurocognitive status and previously identified biomolecular indices of frailty in pre-dementia patients. Muscle biopsies will be collected to assess myocyte contraction and mitochondrial metabolism. Blood and plasma samples will be analysed for laboratory endpoints including nutrition metabolism and omics.

For peer review only

Ethics: issued by the ethical committees of the Azienda Ospedaliera Universitaria Integrata of Verona with respect to scientific content and compliance with applicable research and human subjects' regulation.

Dissemination: Given the broader interest of the society toward undernutrition in elderly, we identify four main target audiences for our research activity: National and local health systems, both internal and external to the project; Targeted population (the elderly); General public; and Academia. These activities include scientific workshops, public health awareness campaigns, project dedicated website and publication is scientific peer-review journals.

Trial registration number: NCT05343611, recruiting.

Strengths and limitations of this study

- This protocol paper presents a novel double-blinded intervention program that focuses on nutrition and exercise for older people with pre-dementia. The program is designed to improve protein energy malnutrition, muscle mass, functional and cognitive abilities.
- The study enables the acquisition of high-quality biological human data to inform the mechanism of muscle mass decline in older people.
- The study and its results are assumed to be applicable among older people as effective intervention strategy.
- Those unable to follow the exercise programme due to disability were not included to the study.

For peer review only

Introduction

Protein-energy malnutrition (PEM) is a condition caused by insufficient intake of protein and energy, and it is a significant concern for older adults. Current data shows that 23% of European older adults are at high risk, and 48% at *some* risk of PEM (1). PEM is a significant risk factor for loss of muscle mass, physical function (i.e., sarcopenia), frailty (1–3) , and cognitive decline (4,5).

Cognitive decline and dementia are commonly linked to a decrease in muscle mass and physical function in older adults. This may be caused by an abnormal regulation of the hypothalamic-pituitary-adrenal (HPA) axis, leading to an alteration of glucose production, and the reduction in energy supply to the muscles. (6). The chronically elevated cortisol level (7,8). Added to this, impaired energy and tissue anabolism pathways, cellular redox and ROS signaling are shown to accumulatively increase tissue degeneration in older people (9). Other possible mechanisms of tissue damage are defective modulation of genes involved in immuno-inflammatory pathways causing low-grade inflammation (10), and hypercortisolism (8).

Combined nutrition and physical exercise interventions are considered a promising strategy to counteract PEM, muscle mass and physical function loss in older age(3,5) but the optimal protocols required are yet to be determined. Dietary protein is a pre-requisite for the maintenance of skeletal muscle mass; stimulating muscle protein synthesis, and it has been shown to overcome anabolic resistance and muscle mass preservation in malnourished older individuals(11). Interestingly, vitamin E supplementation improves myotube survival and prevents myoblasts from atrophy, also stimulating their proliferation and promoting membrane repair during exposure to oxidant challenges which attenuate senescence and replenish the regenerative capacity of these cells(12,13). Further, polyphenols (PP), are ass ociated with inhibition of inflammatory cytokines, oxidative stress, and muscle atrophy-related ubiquitin ligases(14,15). Also, PP supports the activation of insulin growth factor-1 (IGF-1) signalling pathway, mitochondrial biogenesis and function, and differentiation factors involved in myogenesis(14).

A nutritional intervention to counteract PEM should preferably be combined with physical exercise. Previous randomized controlled trials (RCTs) have shown the muscle anabolic exercise effect on muscle mass and strength (16) and on the HPA

For peer review only

axis reactivity and attenuation of hypercortisolism in patients with Alzheimer's Disease (AD) (17). Particularly, the maximal strength training (MST) has previously yielded excellent results in older subjects (18). Also, the high-intensity interval training (HIIT), was previously shown to be excellent for improving health and performance in older subjects (19)and frail patient populations (20). These results suggest that concurrent interventions consisting of both MST and HIIT can induce complementary, beneficial effects on muscle strength, mass, and metabolism (21).The effect of PP or E combined with effective, HIIT, to improve muscle metabolism has not been investigated. Some animal studies have provided evidence that combination of exercise, PP, and vitamins (i.e., vitamin C and E) improved cellular antioxidant enzyme contents and reduced ROS levels (22,23). However, most of the findings obtained from *in vitro* studies and animal models have not been confirmed or fully investigated in clinical trials.

The main hypothesis is the antioxidant and cytoprotective functions of vitamin E (24), combined with the cortisol-lowering effect of chocolate polyphenols (25), can prevent the age-dependent decline of mitochondrial function and nutrient metabolism in skeletal muscle. We will test this hypothesis in a group of older people with stabilized dietary protein intake and physical activity levels. This is important as we are able to account for these two major lifestyle factors and age-dependent muscle function decline before testing the metabolic effects of chocolate polyphenols and vitamin E. The RCT protocol encompass a wide range of clinical and molecular investigations of skeletal muscle mitochondrial function, nutrient metabolism, insulin, and cortisol signaling, omics and tracing the enzymatic and free radical-derived metabolites of vitamin E for the first time in the human muscle.

Methods

Study design

This is a multicenter study, screening for eligible participants will be from 4 centers, Section of Movement Science of the Department of Neuroscience, Biomedicine, and Movement Science of University of Verona, Italy; Centro di Ricerca Sport Montagna e Salute (CeRiSM) – Rovereto, Italy; P. Pederzoli hospital – Peschiera, Italy; and Mons. Mazzali Foundation – Mantua, Italy. The study has been registered with ClinicalTrials.gov Identifier: NCT05343611.

Inclusion Criteria:

For peer review only

Presence of Mild Cognitive Impairment or Mild Dementia. Recruited individuals will be assessed by means of Neuropsychological tests (Mini Mental State Examination, evaluations criteria from Diagnostic and Statistical Manual for Mental Disorder-5) which will be performed by an expert Neuropsychologist.

Exclusion Criteria:

Presence of kidney or liver failure, or any other liver or kidney disease; presence of gastro-intestinal disorders (i.e. irritable bowel syndrome); presence of food intolerance; presence of heart failure, angina, pulmonary disease, cancer and cancer-related cachexia; presence of coagulation disorders; addictive or previous addictive behaviour, defined as the abuse of cannabis, opioids or other drugs, carrier of infectious diseases; presence of musculoskeletal diseases; suffering from mental illness, inability to cooperate; suffering from known cardiac conditions (e.g. pacemakers, arrhythmias, and cardiac conduction disturbances) or peripheral neuropathy; regular users of any proton pump inhibitors (e.g., omeprazole, lansoprazole, pantoprazole), antibiotics, anticoagulant medication or antiplatelet medications in high dose (es: acetylsalicylic acid >200mg x day); mini Mental State (MMSE): results >= 10 points.

Intervention description

After receiving consent, eligible participants will enter the 2-week *Run-in phase*, to stabilize the dietary protein intake at 0.9-1 g/ kg ideal body weight and inform participants about the physical exercise program. Diet quantity and quality will be assessed using a food frequency questionnaire by a dietitian and tailored dietary advice will be provided to maintain the required calorie and protein intake All participants will take part in the supervised physical exercise program consisting of high-intensity aerobic endurance exercise and maximal strength training (MST) three times a week, and each session will last about 50 minutes. The high-intensity aerobic endurance training is carried out walking on a treadmill with 4 x 4 minutes at 85-95% of HRmax, interrupted by 3-minute active recovery periods (~60-70% of HRmax) (26). The MST is carried out in a seated horizontal leg press apparatus and performed as 4 sets with 4 repetitions at ~90% of maximal strength (1RM) (27). Rest periods between the sets will be 3–4 min. The training program will take place at each centre in this multicentre study, where a room will be furnished with all the equipment needed.

For peer review only

Physical exercise sessions will be supervised by a trained research team member and the ratio of supervisor: participants will be 1:2.

After the run-in phase, participants will be randomized into three intervention groups (34 participants in each group). Group A (i.e. control group) will have a high protein diet and take part in the physical exercise programme three times per week, for participants in this arm the diet plan will be adjusted to receive the same overall intake of calories and macronutrients (3 g of proteins, 4 g of carbohydrates, 11 g of fat, 4 g of fibers) that the chocolate products will provide to groups B and C. Group B will receive 30g of 85% dark chocolate high in polyphenols) (polyphenols ≥ 500 mg of polyphenols with ≥ 60 mg of epicatechin) in addition to high protein diet and physical exercise. Group C will receive 30 grams of 85% dark PPs functionalized chocolate with 100 mg vitamin-E per day in addition to high protein diet and physical exercise. On the days of physical exercise for the vitamin-E/ PP groups B and C, chocolate will be consumed in the ninety minutes after the end of the training. The final functionalized chocolate has been produced with a collaborating partner, Nestlè Italiana SPA (owner of "Perugina" brand of pure chocolate products). The concentration of chocolate PP is standardized, and all the patients receive the same dose of chocolate product developed by Perugina Nestlè factory during the first phase of the project (before the initiation for the clinical trial) and thus of chocolate PP that were measured at the Coordinator Unit (Figure 1).

Outcomes

Primary Outcome Measure is the change in free-fat soft tissue mass (g) change in free-fat soft tissue mass, (FFSTM, g), will be assessed by means of a whole-body scan on a dual-energy X- ray absorptiometry scanner. Values at the regional level (upper limbs, lower limbs, and trunk) will be also considered.

Secondary outcome measures are: 1) change in quadriceps volume and cross-sectional area measured by using ultrasound. All ultrasound images will be acquired by an expert operator with the same ultrasound device throughout the whole study using a linear 50 mm transducer; 2) Change in the torque (Nm) and rate of torque development (Nm/s) of quadriceps during Maximal Voluntary Activation and electrically evoked potential Maximal voluntary and electrically evoked muscle contractions of the quadriceps muscle of the dominant leg will be measured utilizing a

For peer review only

custom-made setup; 3) Change in the one repetition maximum load (kg); 4) Change in the Rate of Force Development (N/s); 5) Change in submaximal and maximal oxygen consumption (ml/kg/min) Individuals will perform a 3-speed walking test, on a treadmill. First, the subjects will be asked to stand in resting condition for 2 minutes meanwhile the resting oxygen uptake will be recorded. Then, they will be asked to walk three 5- minute bouts of walking at 80%, 100%, and 120% self-selected speed respectively. Oxygen consumption (ml/kg/min) at these three speeds will be considered for the analysis; 6) Change in Mini-Mental State Examination score (points), the global cognitive functioning will be assessed by means of Mini-Mental State Examination by an expert Neuropsychologist; 7) Change in the Flow-mediated dilation (%) The brachial artery will be imaged using a high-resolution ultrasound Doppler system;8) Change in the Blood flow delta peak (ml/min) during a Single Passive-Leg Movement test; 9) Change in the Pulse Wave Velocity (m/s); 10) Change in distance (meters) during the 6-minute walking test; 11) Change in time (min) during the Time-up and go test (TUG); 12) Change in score (number of raises) during the 30 seconds Chair-stand test; 13) Changes in Circadian Cortisol curve (levels at 4 specific time throughout a day, ng/mL) Salivary cortisol will be measured using plain Sarstedt Salivette collection devices (Nürmbrecht, Germany). Immediately after sample collection, the Salivette tubes will be centrifuged for 2 minutes at 1000 rpm and stored at -80C until analysis. Cortisol levels will be determined by a time-resolved fluorescence immunoassay. To assess the circadian cortisol curve the samples will be taken at 7 am, 11 am, 3 pm and 8 pm;14) Acute Cortisol response to the exercise (delta percentage between before and after a training session, %); 15) Change in IL-6 (pg/mL) and IGF-1 (ng/mL) concentrations; 16) Change in malondialdehyde (MDA, μM); 17) Change in mRNA expression, RNA samples will be processed by following the specific platform protocols and the final results will be bioinformatically analysed. Expression analysis software and pipelines will be used to analyse the differential expression profiles of the selected genes. We will also analyse SUB-NETWORKS, this is to see the relationships that exist between the different transcripts to try and find common molecular pathways; 18) Change in the microbiota composition: Bacterial DNA will be extracted from faecal samples and then amplified and sequenced using a high-throughput next- generation sequencing (NGS) platform able to generate million short sequences (reads) per single run. Sequences will be then processed using a bioinformatic pipeline whose steps that can be summarized as follows: raw data

For peer review only

collection, data cleaning, assembly, gene prediction, taxonomic annotation, gene and protein abundance estimation; 19) Change in the muscle histology and fibre typing; 20) Change in the muscle mitochondrial respiration; 21) Change in the muscle In vitro force characteristics. After the biopsy, fibre bundles of 4–6 mm in length and 0.5 mm in diameter will be dissected from the samples and immersed in skinning solution to which the non-ionic detergent Brij 58 had been added; 23) Change in the muscle single fibre measurements; 24) Change in the muscle cytokine mRNA; 25) Change in the muscle redox status; 26) Change in the muscle proteomics.

A portion of the frozen muscle will be thawed on ice and prepared for proteomic analysis as previously described. A global label-free proteomic approach will be used using an Ultimate 3000 RSLC nano system coupled to a QExactive mass spectrometer. Data analysis will be performed using Proteome Discover and Peaks7.

Clinical and physiological assessments

All assessments are presented in schematic Figure 2 for everyone.

Body composition

Dual X-Ray absorptiometry (DXA) will be used for segmental and total body muscle mass and fat mass and bone mineral density (28). The Quadriceps volume and cross-sectional area (CSA) will be measured by the same ultrasound device throughout the whole study (29).

Physical function tests

6-minute walking test (6MWT), time-up and go (TUG), and 30 seconds chair-stand test. For measuring strength component, individuals will undergo maximal Voluntary Activation (MVC) and electrically evoked potential, to estimate the role of central fatigue; rate of force development (RFD) and peak force will be assessed to provide data on the relative neural and muscular contribution, respectively, to the muscle force development. To assess the energy cost of Walking (Cw) and maximal oxygen consumption. Individuals will perform a 3-speed walking test, on a treadmill.

Cognitive assessment

For peer review only

Patients will undergo a full neuropsychological battery of tests to explore verbal, visuospatial memory, working memory, attention, dual task, and executive functions. The global cognitive functioning will be assessed with MMSE. The Neuropsychiatric Inventory (NPI) will be used to evaluate the presence, frequency, and severity of behavioural disorders. The Geriatric Depression Scale will be used to assess the presence and the severity of depression.

Vascular function

Vascular function will be assessed by means of Flow-mediated dilation (FMD) and single Passive-leg movement (30).

Saliva Cortisol

Cortisol will be collected using salivette with a cotton swab (Sarsted, AG & Co. KG, Nümbrecht, GERMANY) at the beginning and at the end of the three months treatment. Swab samples will be collected: (a) to measure acute response to a single exercise training session, saliva samples will be collected before starting the sessions and right at the end of one of it; (b) to measure the circadian curve, participants will be asked to collect saliva samples in a regular day (i.e. without exercise training) at four specific time points (7am, 11am, 3pm, and 7pm) (31,32).

Blood assays

Whole blood will be examined for glycosylation and transcriptomics. Plasma samples will be obtained for protein oxidation, lipid peroxidation, general hormones, ELISA kit assays. Furthermore, the analysis will include GC-MS and LC-MS/MS determinations of plasma levels of chocolate PPs, and vitamin E metabolites (33). Serum sample will be analyzed for untargeted l metabolomics, this latter is expected to provide a generic fingerprint of the response to the proposed nutritional intervention and to training activities. PBMCs will be used to extract proteins and mRNA for the analysis of genes involved in vitamin E metabolism (such as CYP4F2).

RNA extraction

Total RNA containing miRNA will be isolated by the mirVana miRNA Isolation Kit (Ambion, Austin, TX) according to the manufacturer's directions. RNA concentration and purity will be assessed with 260/280 ratio using a Genequant Pro Classic

For peer review only

spectrophotometer (GE Healthcare, Spain) and RNA integrity by capillary electrophoresis using the RNA 6000 Nano Lab-on-a-Chip kit and the Bioanalyzer 2100 (Agilent Technologies, Santa Clara, CA).

Gene expression profiling

mRNA profiling will be performed using Clariom D-Human Array (Thermo Fisher Scientific). This array comprises >542 000 transcripts constituting over 134 000 gene-level probe sets. Microarray experiments were conducted according to the manufacturer's instructions (Thermo Fisher Scientific). Briefly, 150-ng total RNA will be labelled using GeneChip WT Plus Reagent Kit. The labelling reaction will be hybridized on the array using Hybridization Oven 645 at 45°C for 16 hours. The arrays are stained with Fluidics Station 450 using fluidics script FS450_0001 and then scanned on a GeneChip Scanner 3000 7G. GeneChip Command Console software supplied by Thermo Fisher Scientific will be used to perform gene expression analysis.

Data Analysis of Microarrays

Data (.CEL files) are analyzed and filtered using the Partek Genomic Suite 6.6 software (Partek Inc., St. Louis, MO). Input files are normalized with the RMA algorithm for gene array on core meta probe-sets or miRNAs. A 1-way analysis of variance (ANOVA) will be performed with the Partek Genomics Suite across all samples. Statistically significant small noncoding RNAs and mRNAs between the different groups studied will be identified by using a model ANOVA of $p < .05$. The imported data will be analyzed by principal component analysis (PCA) to determine the significant sources of variability in the data. Differentially expressed genes will be imported into Pathway Studio.

Muscle Biopsy

A sample of skeletal muscle will be obtained with a 13-gauge tru-cut needle from the vastus lateralis muscle. This will be used for the following assays: (A) Assessment of muscle cytokine mRNA for a panel of pro-inflammatory cytokines examined by quantitative real-time PCR (qPCR). RNA will be isolated using standard Trizol® extraction method and purified using RNeasy clean-up kit; cDNA will be synthesised using iScript first strand kit from 1 µg of isolated RNA. (B) Muscle redox status will be undertaken by analysis of the reduced and oxidised glutathione contents of the biopsy

For peer review only

together with analysis of the redox status of mitochondria through analysis of the proportion of peroxiredoxin 3 in the oxidised form, and cytosol by analysis of the proportion of peroxiredoxin 2 in the oxidised form (34) (C) Muscle proteomics: A global label -free proteomic approach will be used at Ultimate 3000 RSLC nano system coupled to a QExactive mass spectrometer. Data analysis will be performed using Proteome Discover and Peaks7 software (34). (D) Determination of mRNA expression: RNA samples will be processed by following the specific platform protocols and the results will be bioinformatically analyzed. Expression analysis software and pipelines will be used to analyse the differential expression profiles of the selected genes. We will also analyse SUB-NETWORKS; this is to see the relationships that exist between the different transcripts to try and find common molecular pathways. (E) Mitochondrial respiration derived from the maximal respiratory capacity (ETS) and in vitro force generation of types 1 and 2 fibers assessed on muscle fibres obtained from the muscle biopsy (35) (F) Targeted metabolomics of chocolate polyphenols and vitamin E by Gas Chromatography Mass Spec and Liquid Chromatography.

Provision of metabolomics

Main analyses will include GC-MS and LC-MS/MS determinations of blood levels of chocolate polyphenols, and vitamin E metabolites, as well as untargeted metabolomics of serum lipids, this latter is expected to provide a generic fingerprint of the response to the proposed nutritional intervention and to HIT. Vitamin E (as alpha-tocopherol) and its long-chain metabolites deriving from CYP450 and free radical dependent biotransformation will be investigated for the first time in human muscle tissue. Moreover, the modification of their levels in this tissue in response to vitamin E supplementation (provided by the innovative food product) is a novel investigation. Bioinformatics analysis will be carried out with the Lipostar software and will provide a model of interpretation of the molecular response and mechanistic effects of the nutritional and physical activity intervention. The model will include the identification of pathways and molecular interactions, as well as of candidate biomarkers of individual and target population response to the proposed intervention.

For peer review only

Sample size

Considering an alpha = 0.05, a power = 0.8 and the 20% of estimated drop out, we aim to recruit 102 subjects (34 in each group). Main outcome is “muscle mass”, and for all the groups treatment duration will be 6 months. In 6 months in the target population the loss of muscle mass is assumed to be 1.0-1.5% (+/- 0.5%) (36). In Control group (Group A), which includes people undergoing targeted exercise, the expected increase is 2% 1.5% (+-0.5%). In treatment groups (Groups B and C) the median average expected increase at second follow-up is 4% 2% (+-0.5%), and 1.5% 4% (+-0.5%) at 6 months (37). The rate of lost at follow-up, derived from previous studies, is 20% (+-2%). Correlation between repeated measures is assumed to be 0.5, variance explained by the between-subjects effect 6.25 and error variance 65. All estimates were performed using Stata v.16.1 (StataCorp LP, College Station, TX, USA) by “power repeated” command.

Allocation and blinding

This is a randomized, double blinded, controlled trial. A blocked randomization approach will be applied to reach the required number for each group. A statistician will create a randomization list including the groups (A, B or C) allocated to each subject, the blocks (after specifying the block size) will be randomly chosen to determine the assignment of all participants. Neither the participants nor research team will know the assignments of the intervention groups including type of chocolate (Vitamin-E and poly phenols, or poly phenols). Also, the participant in the control group (A) will not be aware of the chocolate consumption available for groups B and C.

Patient and Public Involvement

There were no implications for the patient and public involvement.

Statistical analysis

Statistical analysis will be conducted under the supervision of an expert in biostatistics and with the support of LIPOSTAR software. The primary data analysis will be two-way repeated measures ANOVA, including age and gender as covariates, with “time” as the within-group factor and “treatment” as the between-groups factor to calculate difference between groups. In the presence of significant effects, a multiple comparisons tests with Bonferroni’s correction will be performed. The familywise alpha level for significance will be set at 0.05 (two-tails), with Bonferroni’s correction when

For peer review only

needed, for all the analyses. This study is inherently generating extensive clinical and omics data which will be analysed using robust and appropriate data analysis and with machine learning algorithms.

Data monitoring

A log-diary will be kept by each participant and will be checked weekly by the investigators and collaborators. In the diary participants will include information about possible adverse events caused by assessment procedures or related to the diet and training, any important points about the response to the interventions, any possible discomfort experienced during or after the training, or notes regarding diet and supplementation. Prof. Gianluca-Svegliati Baroni of the Gastroenterology Division of the University Hospital of Ancona, Italy will serve as external scientific supervisor of the clinical trial. He is an expert in clinical and preclinical studies of human nutrition and metabolism. He will advise on specific Code: CHOKO-AGE Data: 10/06/2021 Version:1 30 tasks and monitor the different phases of clinical trial from organization to implementation of activities, data gathering and evaluation/interpretation. The quality assurance standards of University of Verona will be adopted to monitor the clinical trial. A delegate of this University will be nominated to perform the monitoring of the different phases of the trial utilizing internal SOPs. The entire set of clinical procedures, operator's activity and collection of experimental data will be verified during a series of visits by the monitor that will occur at the beginning and the end of each time point in the study (Time T00 to T3).

Limitation of the study

There are some limitations to this study, the RCTs population are mostly white European descendant that are not representative of all older population. This study will not be able to account for effect of intervention and change beyond the study duration, making it challenging to evaluate the long-term sustainability of the intervention. By nature, it is not possible to blind participant for exercise intervention, and this may have effect on the participant lifestyle choices beyond what can be captured with the study.

For peer review only

Ethics and disseminations

This protocol and the informed consent forms have been reviewed and approved by the applicable ethical committees of the *Azienda Ospedaliera Universitaria Integrata* of Verona with respect to scientific content and compliance with applicable research and human subjects' regulation. Thereby, this protocol is ethically conducted following the last revision of the Declaration of Helsinki as well as the Declaration of Oviedo. The protocol of the study is designed and will be conducted in order to adhere to Good Clinical Practice principles and procedures and in compliance with the Italian legislation, as described in the following documents and accepted by the investigators of the study with their signature: a) ICH Harmonized Tripartite Guidelines for Good Clinical Practice 1996; b) Directive 91/507/EEC, The Rules Governing Medicinal Products in the European Community; c) D. L.vo n.211 of 24th June 2003; d) D. L.vo n.200 6th November 2007; e) D.M. 21st December 2007. NIH Clinical trial identification ID and number: JPI- ERAHDL NCT05343611.

Consent, confidentiality, declaration of interests

A member of the research team will introduce the trial to individuals who show an interest in taking part in the study and their caregivers. Individuals will also receive information sheets. A member of the research team will discuss the trial with potential participants and their caregivers considering the information provided in the information sheets. Individuals and caregivers will then be able to have an informed discussion with the member of the research team who will obtain written informed consent from the persons willing to participate in the trial. All study-related information will be stored securely at the study site. All participant information will be stored in locked file cabinets in area with limited access. All laboratory specimens, reports, data collection, process and administrative forms will be identified by a coded ID number only to maintain participant confidentiality. All records that contain names or other personnel identifier will be stored separately from study records identified by code number. All local databases will be secured with password-protected access systems. Participants information will be stored for 10 years. Forms, lists, logbook, appointment books, and any other listing that link to participant ID numbers to other identifying information will be stored in a separate, locked file in an area with limited access.

For peer review only

Conflict of Interest

None declared.

Data access

Access to the database to start a data entry session is protected by a username and password. The following precautionary measures are taken to ensure data privacy and to prevent data manipulation and loss: i) Access to data is restricted to authorized members only. The authorized members are the researchers directly involved in the study who will collect, analyze, and interpret the data; ii) The network is protected by a firewall; iii) Internet connection is encrypted with a digital certificate (SSL technology); iv) The database is on a server, password protected, which is changed periodically; v) Access to the database is password protected and is accessible only to those responsible for the center; vi) Periodic backups are performed. vii) and places of conservation (e.g., The paper materials related to clinical evaluations will be stored in cabinets, whose keys will be in possession only of the persons authorized by the persons in charge of the study). Personal data of the subjects will be exclusively accessible for investigators and collaborators/co-investigators, monitors and auditors of the Promoter, and/or for the competent authority, in agreement with what is included in the Informed consent. The Coordinator and the Investigators will inform the patients in a clear and thorough way about the modalities of personal data treatment before their participation to the experiment, as established by the applicable Privacy Laws.

For peer review only

Dissemination policy

A consortium agreement has been established for the dissemination policy. Each Unit is encouraged to publish in written form, oral presentation or making public in any other form (including electronic publication on the internet), hereinafter referred to as the “Publication”, the results of the Project, including but not limited to solely generated Foreground, after providing a copy of the draft Publication to the other Parties at least thirty calendar days prior to submission for publication or disclosure. The other parties may, by giving written notice to the Unit requesting the Publication (a “Confidentiality Notice"), require the publishing Unit to: (a) amend the Publication in order to remove any of its Background that is Confidential Information for the duration of the confidentiality obligation, or to remove any of its Foreground; or (b) amend the Publication if it can reasonably demonstrate that the manuscript compromises

disproportionally its academic or legitimate interests. The other Unit shall give such Confidentiality Notice within thirty calendar days after receipt of the draft Publication. The Confidentiality Notice shall contain specific details to be amended and the Party seeking disclosure shall delete said specific details in as far as they comprise the other Units's Confidential Information from the Publication or amend said specific details as not to Consortium Agreement " Choko-Age project" disproportionally harm the requesting Unit's academic or legitimate interests. If no objection is made within the time limit stated above, the Publication is permitted.

If an objection has been raised the involved Units shall discuss how to overcome the justified grounds for the objection on a timely basis (for example by amendment to the planned Publication before publication). The objecting Unit shall not unreasonably continue the opposition if appropriate actions are performed following the discussion. In any event, any delays of a Publication shall not exceed a period of more than 90 calendar days from the date of the notification from the Unit seeking disclosure. Contributors to the results obtained through work on the Project published shall be named and accredited in the Publication in accordance with academic custom. Other aspects concerning result dissemination and the communications strategy of the project are presented in the "Communication and Dissemination plan" enclosed as Annex IV to the present Agreement.

Author contributions

AP and MI where the co-lead and written the protocol according to the project descriptive, created figures and structured the manuscripts. FG as the PI of the project have contributed equally. All other authors of Choko-Age collaborators all contributed to the conception and design of the work, data acquisition, drafting and revising the study and are accountable for its intellectual content. This is the first version of the protocol submitted to BMJ Open.

Choko-Age Collaborators: Anna Pedrinolla, Masoud Isanejad, Cinzia Antognelli, Desirée Bartolini, Consuelo Borras, Valentina Cavedon, Gabriele Di Sante, Anna Migni, Cristina Mas-Bargues, Chiara Milanese, Claudia Baschirotto, Roberto Modena, Alessandra Pistilli, Mario Rende, Federico Schena , Anna Maria Stabile, Nicola Vincenzo Telesa , Sara Tortorella, Kay Hemmings, Jose Vina , Eivind Wang, Anne McArdle, Malcolm J Jackson, Massimo Venturelli, and Francesco Galli.

For peer review only - http://bmjopen.bmj.com/site/about/guidelines.xhtml

Funding statement

To intervene with high-calory micronutrient-rich diet in elderly patients at risk of PEM could be problematic for different reasons including the difficulty to find palatable food items suitable to satisfy these nutritional criteria. The need for specific innovation in this area of food industry and human nutrition has recently been addressed in the call PREVNUT promoted by the “Joined Programming Initiatives-Healthy Diet for a Healthy Life” (JPI-HDHL) supported by the EU program Horizon 2020 under the umbrella of ERA-Net/ERA-HDHL and the international network of funding organizations reported in (https://www.healthydietforhealthylife.eu/index.php/call-activities/calls/98-calls-site-restyling/583-era-hdhl-2020). This project has received funding from MUR, Ministero dell’Università e della Ricerca (DD17080-24/11/2021) (Italy); Instituto de Salud Carlos III (ISCIII) (Ref. AC20/00026) (Spain); RCN, The Research Council of Norway (Norway) and UKRI, Biotechnology and Biological Sciences Research Council (BBSRC) (grant number BB/V019821/1) (UK), under the umbrella of the European Joint Programming Initiative “A Healthy Diet for a Healthy Life” (JPI HDHL) and of the ERA-NET Cofund ERA-HDHL (GA N° 696295 of the EU Horizon 2020 Research and Innovation Programme).

For peer review only

References

1. Leij-Halfwerk S, Verwijs MH, van Houdt S, Borkent JW, Guaitoli PR, Pelgrim T, et al. Prevalence of protein-energy malnutrition risk in European older adults in community, residential and hospital settings, according to 22 malnutrition screening tools validated for use in adults ≥65 years: A systematic review and meta-analysis. Vol. 126, Maturitas. Elsevier Ireland Ltd; 2019. p. 80–9.

2. Eglseer D, Eminovic S, Lohrmann C. Association between sarcopenia and nutritional status in older adults: A systematic literature review. Vol. 42, Journal of Gerontological Nursing. Slack Incorporated; 2016. p. 33–41.

3. Mathewson SL, Azevedo PS, Gordon AL, Phillips BE, Greig CA. Overcoming protein-energy malnutrition in older adults in the residential care setting: A narrative review of causes and interventions. Ageing Res Rev [Internet]. 2021;70(May):101401. Available from: https://doi.org/10.1016/j.arr.2021.101401

4. Anderson R, Weindruch R. Metabolic reprogramming in dietary restriction. Vol. 35, Interdisciplinary Topics in Gerontology. Interdiscip Top Gerontol; 2007. p. 18–38.

5. Droogsma E, van Asselt D, de Deyn PP. Gewichtsverlust und Unterernährung bei selbstständig lebenden Patienten mit Alzheimer-Demenz: Von populationsbasierten Studien zum klinischen Management. Z Gerontol Geriatr. 2015;48(4):318–24.

6. Martocchia A, Gallucci M, Noale M, Maggi S, Cassol M, Stefanelli M, et al. The increased cortisol levels with preserved rhythmicity in aging and its relationship with dementia and metabolic syndrome. Aging Clin Exp Res. 2022;

7. Venturelli M, Cè E, Limonta E, Muti E, Scarsini R, Brasioli A, et al. Possible Predictors of Involuntary Weight Loss in Patients with Alzheimer's Disease. PLoS One. 2016;

8. Ouanes S, Popp J. High cortisol and the risk of dementia and alzheimer's disease: A review of the literature [Internet]. Vol. 11, Frontiers in Aging Neuroscience. Frontiers Media S.A.; 2019 [cited 2022 Jun 15]. Available from: https://pubmed.ncbi.nlm.nih.gov/30881301/

For peer review only

9. Oudbier SJ, Goh J, Looijaard SMLM, Reijnierse EM, Meskers CGM, Maier AB. Pathophysiological Mechanisms Explaining the Association Between Low Skeletal Muscle Mass and Cognitive Function. The Journals of Gerontology: Series A [Internet]. 2022 Oct 6 [cited 2022 Nov 5];77(10). Available from: https://pubmed.ncbi.nlm.nih.gov/35661882/

10. Beura SK, Dhapola R, Panigrahi AR, Yadav P, Reddy DH, Singh SK. Redefining oxidative stress in Alzheimer’s disease: Targeting platelet reactive oxygen species for novel therapeutic options. Vol. 306, Life Sciences. Elsevier Inc.; 2022.

11. Strasser B, Wolters M, Weyh C, Krüger K, Ticinesi A. The effects of lifestyle and diet on gut microbiota composition, inflammation and muscle performance in our aging society. Nutrients. 2021;13(6).

12. Chung E, Mo H, Wang S, Zu Y, Elfakhani M, Rios SR, et al. Potential roles of vitamin E in age-related changes in skeletal muscle health. Nutrition Research. 2018;49:23–36.

13. Khor SC, Abdul Karim N, Wan Ngah WZ, Mohd Yusof YA, Makpol S. Vitamin e in sarcopenia: Current evidences on its role in prevention and treatment. Oxid Med Cell Longev. 2014;2014.

14. Nikawa T, Ulla A, Sakakibara I. Polyphenols and their effects on muscle atrophy and muscle health. Molecules. 2021;26(16).

15. Thoma A, Akter-Miah T, Reade RL, Lightfoot AP. Targeting reactive oxygen species (ROS) to combat the age-related loss of muscle mass and function. Biogerontology. 2020;21(4):475–84.

16. Wang E, Nyberg SK, Hoff J, Zhao J, Leivseth G, Tørhaug T, et al. Impact of maximal strength training on work efficiency and muscle fiber type in the elderly: Implications for physical function and fall prevention. Exp Gerontol [Internet]. 2017;91:64–71. Available from: http://dx.doi.org/10.1016/j.exger.2017.02.071

17. Pedrinolla A, Schena F, Venturelli M. Resilience to Alzheimer ’ s Disease : The Role of Physical Activity. 2017;546–53.

For peer review only

18. Torvild Kittilsen H, Ingegerdsson Freberg B, Nicolaisen I, Maria Støa E, Bratland-Sanda S, Helgerud J, et al. Responses to Maximal Strength Training in Different Age and Gender Groups. Front Physiol [Internet]. 2021;12:636972. Available from: www.frontiersin.org

19. StØren Ø, Helgerud J, SÆbØ M, StØa EM, Bratland-Sanda S, Unhjem RJ, et al. The Effect of Age on the V-O2max Response to High-Intensity Interval Training. Med Sci Sports Exerc. 2017 Jan 1;49(1):78–85.

20. Helgerud J, Wang E, Mats ·, Mosti P, Øystein ·, Wiggen N, et al. Plantar Xexion training primes peripheral arterial disease patients for improvements in cardiac function. Eur J Appl Physiol. 2009;106:207–15.

21. Mosti MP, Wang E, Wiggen ØN, Helgerud J, Hoff J. Concurrent strength and endurance training improves physical capacity in patients with peripheral arterial disease. Scand J Med Sci Sports [Internet]. 2011;21:e308–14. Available from: https://onlinelibrary.wiley.com/doi/10.1111/j.1600-0838.2011.01294.x

22. Liu S, Meng F, Zhang D, Shi D, Zhou J, Guo S, et al. Lonicera caerulea Berry Polyphenols Extract Alleviates Exercise Fatigue in Mice by Reducing Oxidative Stress, Inflammation, Skeletal Muscle Cell Apoptosis, and by Increasing Cell Proliferation. Front Nutr. 2022;9:853225.

23. Mock JT, Chaudhari K, Sidhu A, Sumien N. The influence of vitamins E and C and exercise on brain aging. Exp Gerontol. 2017;94:69–72.

24. Galli F, Azzi A, Birringer M, Cook-Mills JM, Eggersdorfer M, Frank J, et al. Vitamin E: Emerging aspects and new directions. Vol. 102, Free Radical Biology and Medicine. Elsevier Inc.; 2017. p. 16–36.

25. Ammar A, Mounaturki, Trabelsi K, Bragazzi NL, Boukhris O, Bouaziz M, et al. Effects of natural polyphenol-rich pomegranate juice on the acute and delayed response of Homocysteine and steroidal hormones following weightlifting exercises: A double-blind, placebo-controlled trial. J Int Soc Sports Nutr. 2020 Mar 6;17(1).

For peer review only

26. Brobakken MF, Nyg ard M, Morken G, Reitan SK, Heggelund J, Wang E, et al. Aerobic interval training in standard treatment of out-patients with schizophrenia: a randomized controlled trial. Acta Psychiatr Scand [Internet]. 2019;140:498–507. Available from: https://onlinelibrary.wiley.com/doi/10.1111/acps.13105

27. Tøien T, Unhjem R, Øren TS, Kvellestad ACG, Hoff J, Wang E. Neural Plasticity with Age: Unilateral Maximal Strength Training Augments Efferent Neural Drive to the Contralateral Limb in Older Adults. Journals of Gerontology - Series A Biological Sciences and Medical Sciences. 2018;73(5):596–602.

28. Hart NH, Nimphius S, Spiteri T, Cochrane JL, Newton RU. Segmental musculoskeletal examinations using dual-energy X-ray absorptiometry (DXA): Positioning and analysis considerations. J Sports Sci Med. 2015 Sep 1;14(3):620–6.

29. Franchi M V., Fitze DP, Hanimann J, Sarto F, Spörri J. Panoramic ultrasound vs. MRI for the assessment of hamstrings cross-sectional area and volume in a large athletic cohort. Sci Rep. 2020 Dec 1;10(1).

30. Trinity JD, Kwon OS, Broxterman RM, Gifford JR, Kithas AC, Hydren JR, et al. The role of the endothelium in the hyperemic response to passive leg movement: looking beyond nitric oxide. American Journal of Physiology-Heart and Circulatory Physiology [Internet]. 2020 Dec 11 [cited 2021 Jan 5];ajpheart.00784.2020. Available from: https://journals.physiology.org/doi/10.1152/ajpheart.00784.2020

31. Venturelli M, Amann M, Layec G, Mcdaniel J, Trinity JD, Fjeldstad AS, et al. Passive leg movement-induced hyperaemia with a spinal cord lesion: Evidence of preserved vascular function. Acta Physiologica. 2014 Feb;210(2):429–39.

32. Pedrinolla A, Tamburin S, Brasioli A, Sollima A, Fonte C, Muti E, et al. An Indoor Therapeutic Garden for Behavioral Symptoms in Alzheimer's Disease: A Randomized Controlled Trial. Journal of Alzheimer's Disease. 2019;71(3).

33. Bartolini D, Marinelli R, Giusepponi D, Galarini R, Barola C, Stabile AM, et al. Alpha-tocopherol metabolites (the vitamin E metabolome) and their

For peer review only

interindividual variability during supplementation. Antioxidants. 2021 Feb 1;10(2):1–16.

34. Pugh JN, Stretton C, McDonagh B, Brownridge P, McArdle A, Jackson MJ, et al. Exercise stress leads to an acute loss of mitochondrial proteins and disruption of redox control in skeletal muscle of older subjects: An underlying decrease in resilience with aging? Free Radic Biol Med. 2021 Dec 1;177:88–99.

35. Claflin DR, Larkin LM, Cederna PS, Horowitz JF, Alexander NB, Cole NM, et al. Effects of high-and low-velocity resistance training on the contractile properties of skeletal muscle fibers from young and older humans. J Appl Physiol [Internet]. 2011;111:1021–30. Available from: http://www.jap.org

36. Vikberg S, Sörlén N, Brandén L, Johansson J, Nordström A, Hult A, et al. Effects of Resistance Training on Functional Strength and Muscle Mass in 70-Year-Old Individuals With Pre-sarcopenia: A Randomized Controlled Trial. J Am Med Dir Assoc. 2019;20(1):28–34.

37. Distefano G, Goodpaster BH. Effects of exercise and aging on skeletal muscle. Cold Spring Harb Perspect Med. 2018;8(3).

For peer review only

Figures legends

Figure 1. Study design and Individual's involvement. Flow chart of the study design showing information on enrolment, group assignment, follow-up, and data analysis (panel A) together with the flow chart of individuals involvement (panel B).

Figure 2. Methods of primary, secondary, and explorative outcomes. Primary outcomes include: body composition, specifically free-fat mass in the lower limbs, measured by means of DXA. Another primary outcome consists of maximal voluntary activation measured by MVS+IT+RT technique. Secondary outcomes include cross sectional area of the quadriceps muscle and pinnation angle, salivary cortisol, measured by means of chemiluminescence, and biological outcomes such as: thiols, mRNA and proteomics, vitamin-E, untargeted lipidomic, glycoxidation, hormones, cytokines, epicatechin, lipid peroxidation and protein oxidation. Furthermore, secondary outcomes include mitochondrial respiration measured on muscle fibbers, single fibre force, fibber typing, cytokines, transcriptomics, and proteomic measured in the muscle fibbers. Exploratory outcomes include vitamin-e measured in muscle fibber, and faecal microbiome.

For peer review only

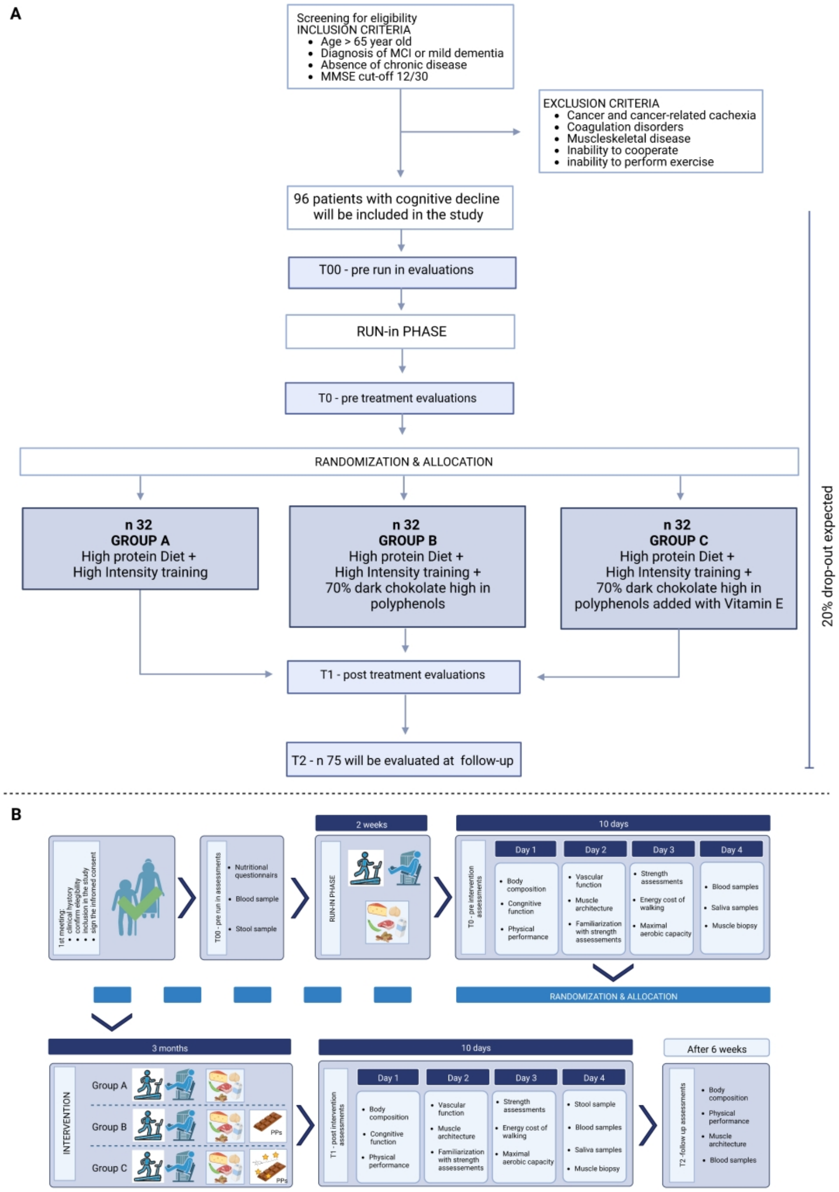

Individual timeline

305x425mm (300 x 300 DPI)

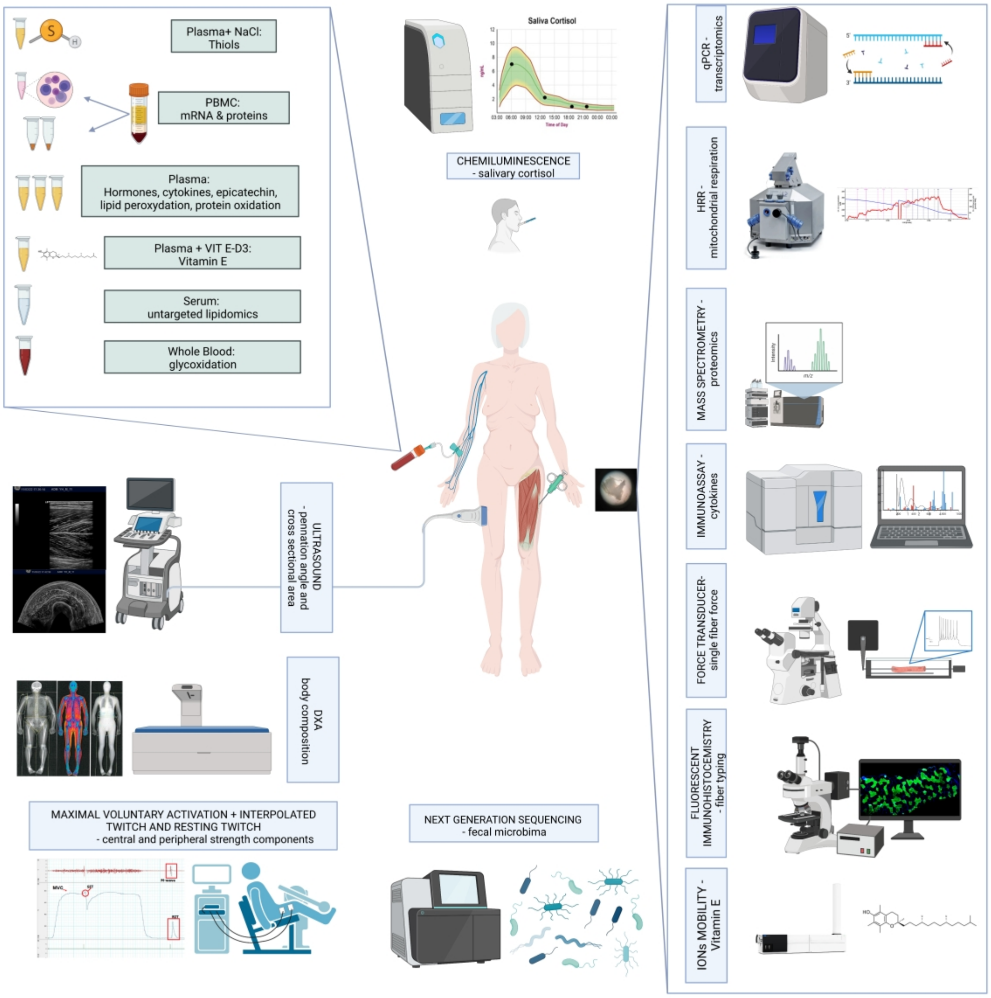

Study graphical abstract for clinical and laboratory endpoints

406x406mm (300 x 300 DPI)

Reporting checklist for protocol of a clinical trial.

Based on the SPIRIT guidelines.

Instructions to authors

Complete this checklist by entering the page numbers from your manuscript where readers will find each of the items listed below.

Your article may not currently address all the items on the checklist. Please modify your text to include the missing information. If you are certain that an item does not apply, please write "n/a" and provide a short explanation.

Upload your completed checklist as an extra file when you submit to a journal.

In your methods section, say that you used the SPIRITreporting guidelines, and cite them as:

Chan A-W, Tetzlaff JM, Gøtzsche PC, Altman DG, Mann H, Berlin J, Dickersin K, Hróbjartsson A, Schulz KF, Parulekar WR, Krleža-Jerić K, Laupacis A, Moher D. SPIRIT 2013 Explanation and Elaboration: Guidance for protocols of clinical trials. BMJ. 2013;346:e7586

		Reporting Item	Page Number
**Administrative information**			
Title	#1	Descriptive title identifying the study design, population, interventions, and, if applicable, trial acronym	1
Trial registration	#2a	Trial identifier and registry name. If not yet registered, name of intended registry	4
Trial registration: data set	#2b	All items from the World Health Organization Trial Registration Data Set	9
Protocol version	#3	Date and version identifier	11
Funding	#4	Sources and types of financial, material, and other support	11
Roles and responsibilities: contributorship	#5a	Names, affiliations, and roles of protocol contributors	10

For peer review only

Roles and responsibilities: sponsor contact information	#5b	Name and contact information for the trial sponsor	NA
Roles and responsibilities: sponsor and funder	#5c	Role of study sponsor and funders, if any, in study design; collection, management, analysis, and interpretation of data; writing of the report; and the decision to submit the report for publication, including whether they will have ultimate authority over any of these activities	11
Roles and responsibilities: committees	#5d	Composition, roles, and responsibilities of the coordinating centre, steering committee, endpoint adjudication committee, data management team, and other individuals or groups overseeing the trial, if applicable (see Item 21a for data monitoring committee)	9
**Introduction**			
Background and rationale	#6a	Description of research question and justification for undertaking the trial, including summary of relevant studies (published and unpublished) examining benefits and harms for each intervention	1
Background and rationale: choice of comparators	#6b	Explanation for choice of comparators	1
Objectives	#7	Specific objectives or hypotheses	2
Trial design	#8	Description of trial design including type of trial (eg, parallel group, crossover, factorial, single group), allocation ratio, and framework (eg, superiority, equivalence, non-inferiority, exploratory)	3
**Methods: Participants, interventions, and outcomes**			
Study setting	#9	Description of study settings (eg, community clinic, academic hospital) and list of countries where data will be collected. Reference to where list of study sites can be	3

For peer review only

		obtained	
Eligibility criteria	#10	Inclusion and exclusion criteria for participants. If applicable, eligibility criteria for study centres and individuals who will perform the interventions (eg, surgeons, psychotherapists)	4
Interventions: description	#11a	Interventions for each group with sufficient detail to allow replication, including how and when they will be administered	4
Interventions: modifications	#11b	Criteria for discontinuing or modifying allocated interventions for a given trial participant (eg, drug dose change in response to harms, participant request, or improving / worsening disease)	4
Interventions: adherance	#11c	Strategies to improve adherence to intervention protocols, and any procedures for monitoring adherence (eg, drug tablet return; laboratory tests)	5
Interventions: concomitant care	#11d	Relevant concomitant care and interventions that are permitted or prohibited during the trial	4
Outcomes	#12	Primary, secondary, and other outcomes, including the specific measurement variable (eg, systolic blood pressure), analysis metric (eg, change from baseline, final value, time to event), method of aggregation (eg, median, proportion), and time point for each outcome. Explanation of the clinical relevance of chosen efficacy and harm outcomes is strongly recommended	5.6
Participant timeline	#13	Time schedule of enrolment, interventions (including any run-ins and washouts), assessments, and visits for participants. A schematic diagram is highly recommended (see Figure)	5
Sample size	#14	Estimated number of participants needed to achieve study objectives and how it was determined, including clinical and statistical assumptions supporting any sample size calculations	8
Recruitment	#15	Strategies for achieving adequate participant enrolment to reach target sample size	8

For peer review only

**Methods: Assignment of interventions (for controlled trials)**			
Allocation: sequence generation	#16a	Method of generating the allocation sequence (eg, computer-generated random numbers), and list of any factors for stratification. To reduce predictability of a random sequence, details of any planned restriction (eg, blocking) should be provided in a separate document that is unavailable to those who enrol participants or assign interventions	8
Allocation concealment mechanism	#16b	Mechanism of implementing the allocation sequence (eg, central telephone; sequentially numbered, opaque, sealed envelopes), describing any steps to conceal the sequence until interventions are assigned	8
Allocation: implementation	#16c	Who will generate the allocation sequence, who will enrol participants, and who will assign participants to interventions	8
Blinding (masking)	#17a	Who will be blinded after assignment to interventions (eg, trial participants, care providers, outcome assessors, data analysts), and how	8
Blinding (masking): emergency unblinding	#17b	If blinded, circumstances under which unblinding is permissible, and procedure for revealing a participant's allocated intervention during the trial	8
**Methods: Data collection, management, and analysis**			
Data collection plan	#18a	Plans for assessment and collection of outcome, baseline, and other trial data, including any related processes to promote data quality (eg, duplicate measurements, training of assessors) and a description of study instruments (eg, questionnaires, laboratory tests) along with their reliability and validity, if known. Reference to where data collection forms can be found, if not in the	9

For peer review only

		protocol	
Data collection plan: retention	#18b	Plans to promote participant retention and complete follow-up, including list of any outcome data to be collected for participants who discontinue or deviate from intervention protocols	9
Data management	#19	Plans for data entry, coding, security, and storage, including any related processes to promote data quality (eg, double data entry; range checks for data values). Reference to where details of data management procedures can be found, if not in the protocol	9
Statistics: outcomes	#20a	Statistical methods for analysing primary and secondary outcomes. Reference to where other details of the statistical analysis plan can be found, if not in the protocol	5
Statistics: additional analyses	#20b	Methods for any additional analyses (eg, subgroup and adjusted analyses)	8
Statistics: analysis population and missing data	#20c	Definition of analysis population relating to protocol non-adherence (eg, as randomised analysis), and any statistical methods to handle missing data (eg, multiple imputation)	8
**Methods: Monitoring**			
Data monitoring: formal committee	#21a	Composition of data monitoring committee (DMC); summary of its role and reporting structure; statement of whether it is independent from the sponsor and competing interests; and reference to where further details about its charter can be found, if not in the protocol. Alternatively, an explanation of why a DMC is not needed	8
Data monitoring: interim analysis	#21b	Description of any interim analyses and stopping guidelines, including who will have access to these interim results and make the final decision to terminate the trial	8
Harms	#22	Plans for collecting, assessing, reporting, and managing solicited and spontaneously reported adverse events and other unintended effects of trial interventions or trial conduct	9
Auditing	#23	Frequency and procedures for auditing trial conduct, if	9

For peer review only

		any, and whether the process will be independent from investigators and the sponsor	
**Ethics and dissemination**			
Research ethics approval	#24	Plans for seeking research ethics committee / institutional review board (REC / IRB) approval	9
Protocol amendments	#25	Plans for communicating important protocol modifications (eg, changes to eligibility criteria, outcomes, analyses) to relevant parties (eg, investigators, REC / IRBs, trial participants, trial registries, journals, regulators)	11
Consent or assent	#26a	Who will obtain informed consent or assent from potential trial participants or authorised surrogates, and how (see Item 32)	9
Consent or assent: ancillary studies	#26b	Additional consent provisions for collection and use of participant data and biological specimens in ancillary studies, if applicable	9
Confidentiality	#27	How personal information about potential and enrolled participants will be collected, shared, and maintained in order to protect confidentiality before, during, and after the trial	9
Declaration of interests	#28	Financial and other competing interests for principal investigators for the overall trial and each study site	9
Data access	#29	Statement of who will have access to the final trial dataset, and disclosure of contractual agreements that limit such access for investigators	9
Ancillary and post trial care	#30	Provisions, if any, for ancillary and post-trial care, and for compensation to those who suffer harm from trial participation	NA
Dissemination policy: trial results	#31a	Plans for investigators and sponsor to communicate trial results to participants, healthcare professionals, the public, and other relevant groups (eg, via publication, reporting in results databases, or other data sharing arrangements), including any publication restrictions	10
Dissemination policy:	#31b	Authorship eligibility guidelines and any intended use of	10

For peer review only

authorship		professional writers	
Dissemination policy: reproducible research	#31c	Plans, if any, for granting public access to the full protocol, participant-level dataset, and statistical code	10

Appendices

Informed consent materials	#32	Model consent form and other related documentation given to participants and authorised surrogates	NA
Biological specimens	#33	Plans for collection, laboratory evaluation, and storage of biological specimens for genetic or molecular analysis in the current trial and for future use in ancillary studies, if applicable	NA

The SPIRIT Explanation and Elaboration paper is distributed under the terms of the Creative Commons Attribution License CC-BY-NC. This checklist was completed on 26. January 2023 using https://www.goodreports.org/, a tool made by the EQUATOR Network in collaboration with Penelope.ai

For peer review only

BMJ Open

Randomized Controlled Trial Combining vitamin E-functionalized Chocolate with physical exercise to reduce the risk of protein-energy malnutrition in pre-dementia Aged people: Study protocol for Choko-Age

Journal:	*BMJ Open*
Manuscript ID	bmjopen-2023-072291.R2
Article Type:	Protocol
Date Submitted by the Author:	07-Nov-2023

Complete List of Authors:	Pedrinolla, Anna ; University of Trento, Cellular, Computational and Integrative Biology<br>Isanejad, Masoud; University of Liverpool, Musculoskeletal & Ageing Science<br>Antognelli, Cinzia ; Università degli Studi di Perugia, Department of Medicine and Surgery<br>Bartolini, Desirée; Università degli Studi di Perugia, Department of Medicine and Surgery, Bioscience and Medical Embryology Division<br>Borras, Consuelo ; University of Valencia, Department of Physiology, Faculty of Medicine<br>Cavedon, Valentina; University of Verona, of the Department of Neuroscience, Biomedicine and Movement (DNBM)<br>Di Sante, Gabriele ; University of Verona, Department of Neurosciences, biomedicine and Movement Sciences<br>Migni, Anna ; University of Perugia, Department of Pharmaceutical Sciences, Lipidomics and Micronutrient<br>Mas-Bargues, Cristina ; University of Valencia, Freshage Research Group, Department of Physiology, Faculty of Medicine, University of Valencia, Centro de Investigación Biomédica en Red Fragilidad y Envejecimiento Saludable-Instituto de Salud Carlos III (CIBERFES-ISCIII)<br>Milanese, Chiara ; University of Verona, Department of Neuroscience, Biomedicine and Movement Sciences<br>Baschirotto, Claudia ; University of Verona, Department of Neurosciences, biomedicine and Movement Sciences<br>Modena, Roberto ; Molde University College, Department of Health and Social Sciences<br>Pistilli , Alessandra ; University of Verona, of the Department of Neuroscience, Biomedicine and Movement<br>Rende , Mario ; University of Verona, of the Department of Neuroscience, Biomedicine and Movement (DNBM)<br>Schena, Federico; University of Verona, Department of Neuroscience, Biomedicine and Movement Sciences<br>Stabile , Anna Maria; University of Verona, of the Department of Neuroscience, Biomedicine and Movement (DNBM)<br>Vincenzo Telesa, Nicola; University of Perugia, Department of Medicine and Surgery<br>Tortorella, Sara ; University of Perugia

	Hemmings , Kay ; University of Liverpool, Musculoskeletal & Ageing Science<br>Vina, Jose; University of Valencia, Department of Physiology, Faculty of Medicine<br>Wang , Eivind ; Molde University College, Department of Health and Social Sciences; St Olavs Hospital Trondheim University Hospital<br>McArdle, Anne ; University of Liverpool Institute of Ageing and Chronic Disease, MRC-Versus Arthritis Centre for Integrated Research into Musculoskeletal Ageing (CIMA), Musculoskeletal & Ageing Science,<br>Jackson, Malcolm; University of Liverpool, MRC-Versus Arthritis Centre for Integrated Research into Musculoskeletal Ageing (CIMA), Musculoskeletal & Ageing Science<br>Venturelli , Massimo ; University of Verona, of the Department of Neuroscience, Biomedicine and Movement<br>Galli, Francesco; University of Perugia, Department of Pharmaceutical Sciences
**Primary Subject Heading**:	Nutrition and metabolism
Secondary Subject Heading:	Complementary medicine
Keywords:	Dementia, Musculoskeletal disorders < ORTHOPAEDIC & TRAUMA SURGERY, NUTRITION & DIETETICS, Neuromuscular disease < NEUROLOGY, Aged

SCHOLARONE™
Manuscripts

Randomized Controlled Trial Combining Vitamin E-functionalized Chocolate with physical exercise to reduce the risk of protein-energy malnutrition in pre-dementia Aged people: Study protocol for Choko-Age

Anna Pedrinolla[1*] & Masoud Isanejad[2*], Cinzia Antognelli[3], Desirée Bartolini[4], Consuelo Borras[5], Valentina Cavedon[6], Gabriele Di Sante[7], Anna Migni[4], Cristina Mas-Bargues[5], Chiara Milanese[6], Claudia Baschirotto[6], Roberto Modena[8], Alessandra Pistilli [6], Mario Rende [6], Federico Schena [6], Anna Maria Stabile [6], Nicola Vincenzo Telesa [3], Sara Tortorella[9], Kay Hemmings [2] Jose Vina [5], Eivind Wang [8,10], Anne McArdle [2], Malcolm J Jackson[2], Massimo Venturelli [6], Francesco Galli

[1]Cellular, Computational and Integrative Biology – CIBIO, University of Trento, Trento, Italy.

[2] MRC-Versus Arthritis Centre for Integrated Research into Musculoskeletal Ageing (CIMA), Department of Musculoskeletal and Ageing Science, Institute of Life Course and Medical Sciences, University of Liverpool, Liverpool L7 8TX, U.K.

[3] Department of Medicine and Surgery, Bioscience and Medical Embryology Division. P.le L. Severi 1 06132 Perugia, Italy

[4] Department of Pharmaceutical Sciences, Lipidomics and Micronutrient Vitamins Lab. and Human Anatomy Lab., University of Perugia, 06126 Perugia, Italy.

[5] Freshage Research Group, Department of Physiology, Faculty of Medicine, University of Valencia, Centro de Investigación Biomédica en Red Fragilidad y Envejecimiento Saludable-Instituto de Salud Carlos III (CIBERFES-ISCIII), INCLIVA, Valencia, 46010, Spain

[6] Department of Neurosciences, biomedicine and Movement Sciences, Univeristy of Verona, Verona, Italy

[7]Department of Medicine and Surgery, Section of Human, Clinical and Forensic Anatomy. P.le L. Severi 1 06132 Perugia

[8] Department of Health and Social Sciences, Molde University College, Norway

[9] Molecular Horizon s.r.l., Via Montelino 30, 06084 Bettona, Italy.

For peer review only

For peer review only - http://bmjopen.bmj.com/site/about/guidelines.xhtml

[10] Department of Østmarka, Division of Mental Health Care, St. Olavs Hospital, Trondheim University Hospital, Trondheim, Norway

Corresponding authors: Anna Pedrinolla (anna.pedrinolla@univr.it) and Masoud Isanejad (masoud.isanejad@liverpool.ac.uk) contributed equally to this work.

Keywords: Vitamin E, Polyphenols, Malnutrition, Exercise, Sarcopenia, Dementia

Word Count: 6265

Abstract

Objective: protein energy malnutrition (PEM) and the subsequent muscle wasting (sarcopenia) are common ageing complications. It is knowing to be also associated with dementia. Our programme will test the cytoprotective functions of vitamin E combined with the cortisol lowering effect of chocolate polyphenols, in combination with muscle anabolic effect of adequate dietary protein intake and physical exercise to prevent the age-dependent decline of muscle mass and its key underpinning mechanisms including mitochondrial function, and nutrient metabolism in muscle in the elderly.

Methods and analysis: in 2020, a 6-month double-blind randomized controlled trial in 75 pre-dementia older people was launched to prevent muscle mass loss, in respond to the 'Joint Programming Initiative A healthy diet for a healthy life'. In the run-in phase participants will be stabilized on protein rich diet (0.9-1.0 g protein/Kg ideal body weight/day) and physical exercise program (High Intensity Interval Training specifically developed for these subjects). Subsequently they will be randomized in to 3 groups (1:1:1). The study arms will have similar isocaloric diet and follow similar physical exercise programme. Control group (n=25) will maintain the baseline diet; intervention groups will consume either 30 g/day of dark chocolate containing 500 mg total polyphenols (corresponding to 60 mg epicatechin) and 100 mg vitamin E (as RRR-alpha-tocopherol) (n=25); or the high polyphenol chocolate without additional vitamin E (n=25). Muscle mass will be the primary endpoint. Other outcomes are neurocognitive status and previously identified biomolecular indices of frailty in pre-dementia patients. Muscle biopsies will be collected to assess myocyte contraction and mitochondrial metabolism. Blood and plasma samples will be analysed for laboratory endpoints including nutrition metabolism and omics.

For peer review only

Ethics and dissemination: all the ethical and regulatory approvals have been obtained by the ethical committees of the Azienda Ospedaliera Universitaria Integrata of Verona with respect to scientific content and compliance with applicable research and human subjects' regulation. Given the broader interest of the society toward undernutrition in elderly, we identify four main target audiences for our research activity: National and local health systems, both internal and external to the project; Targeted population (the elderly); General public; and Academia. These activities include scientific workshops, public health awareness campaigns, project dedicated website and publication is scientific peer-review journals.

Trial registration number: NCT05343611, recruiting.

Strengths and limitations of this study

- A novel polyphenol and vitamin E combined with exercise intervention programme in pre-dementia to improve muscle health.
- .
- The program is designed to improve protein energy malnutrition, muscle mass, functional and cognitive abilities.
- The study enables the acquisition of high-quality biological human data to inform the mechanism of muscle mass decline in older people.The study and its results are assumed to be applicable among older people as effective intervention strategy.
- A limitation to this study was, those unable to follow the exercise programme due to disability were not included to the study.
- Study did not have prior patient and public involvement.

For peer review only

Introduction

Protein-energy malnutrition (PEM) is a condition caused by insufficient intake of protein and energy, and it is a significant concern for older adults. Current data shows that 23% of European older adults are at high risk, and 48% at *some* risk of PEM (1). PEM is a significant risk factor for loss of muscle mass, physical function (i.e., sarcopenia), frailty (1–3) , and cognitive decline (4,5).

Cognitive decline and dementia are commonly linked to a decrease in muscle mass and physical function in older adults. This may be caused by an abnormal regulation of the hypothalamic-pituitary-adrenal (HPA) axis, leading to an alteration of glucose production, and the reduction in energy supply to the muscles. (6). The chronically elevated cortisol level (7,8). Added to this, impaired energy and tissue anabolism pathways, cellular redox and ROS signaling are shown to accumulatively increase tissue degeneration in older people (9). Other possible mechanisms of tissue damage are defective modulation of genes involved in immuno-inflammatory pathways causing low-grade inflammation (10), and hypercortisolism (8).

Combined nutrition and physical exercise interventions are considered a promising strategy to counteract PEM, muscle mass and physical function loss in older age(3,5) but the optimal protocols required are yet to be determined. Dietary protein is a pre-requisite for the maintenance of skeletal muscle mass; stimulating muscle protein synthesis, and it has been shown to overcome anabolic resistance and muscle mass preservation in malnourished older individuals(11). Interestingly, vitamin E supplementation improves myotube survival and prevents myoblasts from atrophy, also stimulating their proliferation and promoting membrane repair during exposure to oxidant challenges which attenuate senescence and replenish the regenerative capacity of these cells(12,13). Further, polyphenols (PP), are ass ociated with inhibition of inflammatory cytokines, oxidative stress, and muscle atrophy-related ubiquitin ligases(14,15). Also, PP supports the activation of insulin growth factor-1 (IGF-1) signalling pathway, mitochondrial biogenesis and function, and differentiation factors involved in myogenesis(14).

A nutritional intervention to counteract PEM should preferably be combined with physical exercise. Previous randomized controlled trials (RCTs) have shown the muscle anabolic exercise effect on muscle mass and strength (16) and on the HPA

For peer review only

axis reactivity and attenuation of hypercortisolism in patients with Alzheimer's Disease (AD) (17). Particularly, the maximal strength training (MST) has previously yielded excellent results in older subjects (18). Also, the high-intensity interval training (HIIT), was previously shown to be excellent for improving health and performance in older subjects (19)and frail patient populations (20). These results suggest that concurrent interventions consisting of both MST and HIIT can induce complementary, beneficial effects on muscle strength, mass, and metabolism (21).The effect of PP or E combined with effective, HIIT, to improve muscle metabolism has not been investigated. Some animal studies have provided evidence that combination of exercise, PP, and vitamins (i.e., vitamin C and E) improved cellular antioxidant enzyme contents and reduced ROS levels (22,23). However, most of the findings obtained from *in vitro* studies and animal models have not been confirmed or fully investigated in clinical trials.

The main hypothesis is the antioxidant and cytoprotective functions of vitamin E (24), combined with the cortisol-lowering effect of chocolate polyphenols (25), can prevent the age-dependent decline of mitochondrial function and nutrient metabolism in skeletal muscle. We will test this hypothesis in a group of older people with stabilized dietary protein intake and physical activity levels. This is important as we are able to account for these two major lifestyle factors and age-dependent muscle function decline before testing the metabolic effects of chocolate polyphenols and vitamin E. The RCT protocol encompass a wide range of clinical and molecular investigations of skeletal muscle mitochondrial function, nutrient metabolism, insulin, and cortisol signaling, omics and tracing the enzymatic and free radical-derived metabolites of vitamin E for the first time in the human muscle.

Methods

Study design

This is a multicenter study, screening for eligible participants will be from 4 centers, Section of Movement Science of the Department of Neuroscience, Biomedicine, and Movement Science of University of Verona, Italy; Centro di Ricerca Sport Montagna e Salute (CeRiSM) – Rovereto, Italy; P. Pederzoli hospital – Peschiera, Italy; and Mons. Mazzali Foundation – Mantua, Italy. The study has been registered with ClinicalTrials.gov Identifier: NCT05343611.

Inclusion Criteria:

For peer review only

Presence of Mild Cognitive Impairment or Mild Dementia. Recruited individuals will be assessed by means of Neuropsychological tests (Mini Mental State Examination, evaluations criteria from Diagnostic and Statistical Manual for Mental Disorder-5) which will be performed by an expert Neuropsychologist.

Exclusion Criteria:

Presence of kidney or liver failure, or any other liver or kidney disease; presence of gastro-intestinal disorders (i.e. irritable bowel syndrome); presence of food intolerance; presence of heart failure, angina, pulmonary disease, cancer and cancer-related cachexia; presence of coagulation disorders; addictive or previous addictive behaviour, defined as the abuse of cannabis, opioids or other drugs, carrier of infectious diseases; presence of musculoskeletal diseases; suffering from mental illness, inability to cooperate; suffering from known cardiac conditions (e.g. pacemakers, arrhythmias, and cardiac conduction disturbances) or peripheral neuropathy; regular users of any proton pump inhibitors (e.g., omeprazole, lansoprazole, pantoprazole), antibiotics, anticoagulant medication or antiplatelet medications in high dose (es: acetylsalicylic acid >200mg x day); mini Mental State (MMSE): results >= 10 points.

Intervention description

After receiving consent, eligible participants will enter the 2-week *Run-in phase*, to stabilize the dietary protein intake at 0.9-1 g/ kg ideal body weight and inform participants about the physical exercise program. Diet quantity and quality will be assessed using a food frequency questionnaire by a dietitian and tailored dietary advice will be provided to maintain the required calorie and protein intake All participants will take part in the supervised physical exercise program consisting of high-intensity aerobic endurance exercise and maximal strength training (MST) three times a week, and each session will last about 50 minutes. The high-intensity aerobic endurance training is carried out walking on a treadmill with 4 x 4 minutes at 85-95% of HRmax, interrupted by 3-minute active recovery periods (~60-70% of HRmax) (26). The MST is carried out in a seated horizontal leg press apparatus and performed as 4 sets with 4 repetitions at ~90% of maximal strength (1RM) (27). Rest periods between the sets will be 3–4 min. The training program will take place at each centre in this multicentre study, where a room will be furnished with all the equipment needed.

For peer review only

Physical exercise sessions will be supervised by a trained research team member and the ratio of supervisor: participants will be 1:2.

After the run-in phase, participants will be randomized into three intervention groups (34 participants in each group). Group A (i.e. control group) will have a high protein diet and take part in the physical exercise programme three times per week, for participants in this arm the diet plan will be adjusted to receive the same overall intake of calories and macronutrients (3 g of proteins, 4 g of carbohydrates, 11 g of fat, 4 g of fibers) that the chocolate products will provide to groups B and C. Group B will receive 30g of 85% dark chocolate high in polyphenols) (polyphenols ≥ 500 mg of polyphenols with ≥ 60 mg of epicatechin) in addition to high protein diet and physical exercise. Group C will receive 30 grams of 85% dark PPs functionalized chocolate with 100 mg vitamin-E per day in addition to high protein diet and physical exercise. On the days of physical exercise for the vitamin-E/ PP groups B and C, chocolate will be consumed in the ninety minutes after the end of the training. The final functionalized chocolate has been produced with a collaborating partner, Nestlè Italiana SPA (owner of “Perugina” brand of pure chocolate products). The concentration of chocolate PP is standardized, and all the patients receive the same dose of chocolate product developed by Perugina Nestlè factory during the first phase of the project (before the initiation for the clinical trial) and thus of chocolate PP that were measured at the Coordinator Unit (Figure 1).

Outcomes

Primary Outcome Measure is the change in free-fat soft tissue mass (g) change in free-fat soft tissue mass, (FFSTM, g), will be assessed by means of a whole-body scan on a dual-energy X- ray absorptiometry scanner. Values at the regional level (upper limbs, lower limbs, and trunk) will be also considered.

Secondary outcome measures are: 1) change in quadriceps volume and cross-sectional area measured by using ultrasound. All ultrasound images will be acquired by an expert operator with the same ultrasound device throughout the whole study using a linear 50 mm transducer; 2) Change in the torque (Nm) and rate of torque development (Nm/s) of quadriceps during Maximal Voluntary Activation and electrically evoked potential Maximal voluntary and electrically evoked muscle contractions of the quadriceps muscle of the dominant leg will be measured utilizing a

For peer review only

custom-made setup; 3) Change in the one repetition maximum load (kg); 4) Change in the Rate of Force Development (N/s); 5) Change in submaximal and maximal oxygen consumption (ml/kg/min) Individuals will perform a 3-speed walking test, on a treadmill. First, the subjects will be asked to stand in resting condition for 2 minutes meanwhile the resting oxygen uptake will be recorded. Then, they will be asked to walk three 5- minute bouts of walking at 80%, 100%, and 120% self-selected speed respectively. Oxygen consumption (ml/kg/min) at these three speeds will be considered for the analysis; 6) Change in Mini-Mental State Examination score (points), the global cognitive functioning will be assessed by means of Mini-Mental State Examination by an expert Neuropsychologist; 7) Change in the Flow-mediated dilation (%) The brachial artery will be imaged using a high-resolution ultrasound Doppler system;8) Change in the Blood flow delta peak (ml/min) during a Single Passive-Leg Movement test; 9) Change in the Pulse Wave Velocity (m/s); 10) Change in distance (meters) during the 6-minute walking test; 11) Change in time (min) during the Time-up and go test (TUG); 12) Change in score (number of raises) during the 30 seconds Chair-stand test; 13) Changes in Circadian Cortisol curve (levels at 4 specific time throughout a day, ng/mL) Salivary cortisol will be measured using plain Sarstedt Salivette collection devices (Nürmbrecht, Germany). Immediately after sample collection, the Salivette tubes will be centrifuged for 2 minutes at 1000 rpm and stored at -80C until analysis. Cortisol levels will be determined by a time-resolved fluorescence immunoassay. To assess the circadian cortisol curve the samples will be taken at 7 am, 11 am, 3 pm and 8 pm;14) Acute Cortisol response to the exercise (delta percentage between before and after a training session, %); 15) Change in IL-6 (pg/mL) and IGF-1 (ng/mL) concentrations; 16) Change in malondialdehyde (MDA, μM); 17) Change in mRNA expression, RNA samples will be processed by following the specific platform protocols and the final results will be bioinformatically analysed. Expression analysis software and pipelines will be used to analyse the differential expression profiles of the selected genes. We will also analyse SUB-NETWORKS, this is to see the relationships that exist between the different transcripts to try and find common molecular pathways; 18) Change in the microbiota composition: Bacterial DNA will be extracted from faecal samples and then amplified and sequenced using a high-throughput next- generation sequencing (NGS) platform able to generate million short sequences (reads) per single run. Sequences will be then processed using a bioinformatic pipeline whose steps that can be summarized as follows: raw data

For peer review only

collection, data cleaning, assembly, gene prediction, taxonomic annotation, gene and protein abundance estimation; 19) Change in the muscle histology and fibre typing; 20) Change in the muscle mitochondrial respiration; 21) Change in the muscle In vitro force characteristics. After the biopsy, fibre bundles of 4–6 mm in length and 0.5 mm in diameter will be dissected from the samples and immersed in skinning solution to which the non-ionic detergent Brij 58 had been added; 23) Change in the muscle single fibre measurements; 24) Change in the muscle cytokine mRNA; 25) Change in the muscle redox status; 26) Change in the muscle proteomics.

A portion of the frozen muscle will be thawed on ice and prepared for proteomic analysis as previously described. A global label-free proteomic approach will be used using an Ultimate 3000 RSLC nano system coupled to a QExactive mass spectrometer. Data analysis will be performed using Proteome Discover and Peaks7.

Clinical and physiological assessments

All assessments are presented in schematic Figure 2 for everyone.

Body composition

Dual X-Ray absorptiometry (DXA) will be used for segmental and total body muscle mass and fat mass and bone mineral density (28). The Quadriceps volume and cross-sectional area (CSA) will be measured by the same ultrasound device throughout the whole study (29).

Physical function tests

6-minute walking test (6MWT), time-up and go (TUG), and 30 seconds chair-stand test. For measuring strength component, individuals will undergo maximal Voluntary Activation (MVC) and electrically evoked potential, to estimate the role of central fatigue; rate of force development (RFD) and peak force will be assessed to provide data on the relative neural and muscular contribution, respectively, to the muscle force development. To assess the energy cost of Walking (Cw) and maximal oxygen consumption. Individuals will perform a 3-speed walking test, on a treadmill.

Cognitive assessment

For peer review only

Patients will undergo a full neuropsychological battery of tests to explore verbal, visuospatial memory, working memory, attention, dual task, and executive functions. The global cognitive functioning will be assessed with MMSE. The Neuropsychiatric Inventory (NPI) will be used to evaluate the presence, frequency, and severity of behavioural disorders. The Geriatric Depression Scale will be used to assess the presence and the severity of depression.

Vascular function

Vascular function will be assessed by means of Flow-mediated dilation (FMD) and single Passive-leg movement (30).

Saliva Cortisol

Cortisol will be collected using salivette with a cotton swab (Sarsted, AG & Co. KG, Nümbrecht, GERMANY) at the beginning and at the end of the three months treatment. Swab samples will be collected: (a) to measure acute response to a single exercise training session, saliva samples will be collected before starting the sessions and right at the end of one of it; (b) to measure the circadian curve, participants will be asked to collect saliva samples in a regular day (i.e. without exercise training) at four specific time points (7am, 11am, 3pm, and 7pm) (31,32).

Blood assays

Whole blood will be examined for glycosylation and transcriptomics. Plasma samples will be obtained for protein oxidation, lipid peroxidation, general hormones, ELISA kit assays. Furthermore, the analysis will include GC-MS and LC-MS/MS determinations of plasma levels of chocolate PPs, and vitamin E metabolites (33). Serum sample will be analyzed for untargeted l metabolomics, this latter is expected to provide a generic fingerprint of the response to the proposed nutritional intervention and to training activities. PBMCs will be used to extract proteins and mRNA for the analysis of genes involved in vitamin E metabolism (such as CYP4F2).

RNA extraction

Total RNA containing miRNA will be isolated by the mirVana miRNA Isolation Kit (Ambion, Austin, TX) according to the manufacturer's directions. RNA concentration and purity will be assessed with 260/280 ratio using a Genequant Pro Classic

For peer review only

spectrophotometer (GE Healthcare, Spain) and RNA integrity by capillary electrophoresis using the RNA 6000 Nano Lab-on-a-Chip kit and the Bioanalyzer 2100 (Agilent Technologies, Santa Clara, CA).

Gene expression profiling

mRNA profiling will be performed using Clariom D-Human Array (Thermo Fisher Scientific). This array comprises >542 000 transcripts constituting over 134 000 gene-level probe sets. Microarray experiments were conducted according to the manufacturer's instructions (Thermo Fisher Scientific). Briefly, 150-ng total RNA will be labelled using GeneChip WT Plus Reagent Kit. The labelling reaction will be hybridized on the array using Hybridization Oven 645 at 45°C for 16 hours. The arrays are stained with Fluidics Station 450 using fluidics script FS450_0001 and then scanned on a GeneChip Scanner 3000 7G. GeneChip Command Console software supplied by Thermo Fisher Scientific will be used to perform gene expression analysis.

Data Analysis of Microarrays

Data (.CEL files) are analyzed and filtered using the Partek Genomic Suite 6.6 software (Partek Inc., St. Louis, MO). Input files are normalized with the RMA algorithm for gene array on core meta probe-sets or miRNAs. A 1-way analysis of variance (ANOVA) will be performed with the Partek Genomics Suite across all samples. Statistically significant small noncoding RNAs and mRNAs between the different groups studied will be identified by using a model ANOVA of $p < .05$. The imported data will be analyzed by principal component analysis (PCA) to determine the significant sources of variability in the data. Differentially expressed genes will be imported into Pathway Studio.

Muscle Biopsy

A sample of skeletal muscle will be obtained with a 13-gauge tru-cut needle from the vastus lateralis muscle. This will be used for the following assays: (A) Assessment of muscle cytokine mRNA for a panel of pro-inflammatory cytokines examined by quantitative real-time PCR (qPCR). RNA will be isolated using standard Trizol® extraction method and purified using RNeasy clean-up kit; cDNA will be synthesised using iScript first strand kit from 1 µg of isolated RNA. (B) Muscle redox status will be undertaken by analysis of the reduced and oxidised glutathione contents of the biopsy

For peer review only

together with analysis of the redox status of mitochondria through analysis of the proportion of peroxiredoxin 3 in the oxidised form, and cytosol by analysis of the proportion of peroxiredoxin 2 in the oxidised form (34) (C) Muscle proteomics: A global label -free proteomic approach will be used at Ultimate 3000 RSLC nano system coupled to a QExactive mass spectrometer. Data analysis will be performed using Proteome Discover and Peaks7 software (34). (D) Determination of mRNA expression: RNA samples will be processed by following the specific platform protocols and the results will be bioinformatically analyzed. Expression analysis software and pipelines will be used to analyse the differential expression profiles of the selected genes. We will also analyse SUB-NETWORKS; this is to see the relationships that exist between the different transcripts to try and find common molecular pathways. (E) Mitochondrial respiration derived from the maximal respiratory capacity (ETS) and in vitro force generation of types 1 and 2 fibers assessed on muscle fibres obtained from the muscle biopsy (35) (F) Targeted metabolomics of chocolate polyphenols and vitamin E by Gas Chromatography Mass Spec and Liquid Chromatography.

Provision of metabolomics

Main analyses will include GC-MS and LC-MS/MS determinations of blood levels of chocolate polyphenols, and vitamin E metabolites, as well as untargeted metabolomics of serum lipids, this latter is expected to provide a generic fingerprint of the response to the proposed nutritional intervention and to HIT. Vitamin E (as alpha-tocopherol) and its long-chain metabolites deriving from CYP450 and free radical dependent biotransformation will be investigated for the first time in human muscle tissue. Moreover, the modification of their levels in this tissue in response to vitamin E supplementation (provided by the innovative food product) is a novel investigation. Bioinformatics analysis will be carried out with the Lipostar software and will provide a model of interpretation of the molecular response and mechanistic effects of the nutritional and physical activity intervention. The model will include the identification of pathways and molecular interactions, as well as of candidate biomarkers of individual and target population response to the proposed intervention.

For peer review only

Sample size

Considering an alpha = 0.05, a power = 0.8 and the 20% of estimated drop out, we aim to recruit 102 subjects (34 in each group). Main outcome is “muscle mass”, and for all the groups treatment duration will be 6 months. In 6 months in the target population the loss of muscle mass is assumed to be 1.0-1.5% (+/- 0.5%) (36). In Control group (Group A), which includes people undergoing targeted exercise, the expected increase is 2% 1.5% (+-0.5%). In treatment groups (Groups B and C) the median average expected increase at second follow-up is 4% 2% (+-0.5%), and 1.5% 4% (+-0.5%) at 6 months (37). The rate of lost at follow-up, derived from previous studies, is 20% (+-2%). Correlation between repeated measures is assumed to be 0.5, variance explained by the between-subjects effect 6.25 and error variance 65. All estimates were performed using Stata v.16.1 (StataCorp LP, College Station, TX, USA) by “power repeated” command.

Allocation and blinding

This is a randomized, double blinded, controlled trial. A blocked randomization approach will be applied to reach the required number for each group. A statistician will create a randomization list including the groups (A, B or C) allocated to each subject, the blocks (after specifying the block size) will be randomly chosen to determine the assignment of all participants. Neither the participants nor research team will know the assignments of the intervention groups including type of chocolate (Vitamin-E and poly phenols, or poly phenols). Also, the participant in the control group (A) will not be aware of the chocolate consumption available for groups B and C.

Patient and Public Involvement

There were no implications for the patient and public involvement.

Statistical analysis

Statistical analysis will be conducted under the supervision of an expert in biostatistics and with the support of LIPOSTAR software. The primary data analysis will be two-way repeated measures ANOVA, including age and gender as covariates, with “time” as the within-group factor and “treatment” as the between-groups factor to calculate difference between groups. In the presence of significant effects, a multiple comparisons tests with Bonferroni’s correction will be performed.

For peer review only

We will also employ mixed model analysis to account for both fixed effects, and random effects, to capture variability or correlations within the data that are not under our direct control. Also, since we are dealing with multiple sources of variation, such as repeated measurements on the same subjects, nested or hierarchical data structures, and potentially correlated factors. Mixed model analysis can provide a more accurate and robust understanding of the relationships within our data.

The familywise alpha level for significance will be set at 0.05 (two-tails), with Bonferroni's correction when needed, for all the analyses. This study is inherently generating extensive clinical and omics data which will be analysed using robust and appropriate data analysis and with machine learning algorithms.

Data monitoring

A log-diary will be kept by each participant and will be checked weekly by the investigators and collaborators. In the diary participants will include information about possible adverse events caused by assessment procedures or related to the diet and training, any important points about the response to the interventions, any possible discomfort experienced during or after the training, or notes regarding diet and supplementation. Prof. Gianluca-Svegliati Baroni of the Gastroenterology Division of the University Hospital of Ancona, Italy will serve as external scientific supervisor of the clinical trial. He is an expert in clinical and preclinical studies of human nutrition and metabolism. He will advise on specific Code: CHOKO-AGE Data: 10/06/2021 Version:1 30 tasks and monitor the different phases of clinical trial from organization to implementation of activities, data gathering and evaluation/interpretation. The quality assurance standards of University of Verona will be adopted to monitor the clinical trial. A delegate of this University will be nominated to perform the monitoring of the different phases of the trial utilizing internal SOPs. The entire set of clinical procedures, operator's activity and collection of experimental data will be verified during a series of visits by the monitor that will occur at the beginning and the end of each time point in the study (Time T00 to T3).

Limitation of the study

There are some limitations to this study, the RCTs population are mostly white European descendant that are not representative of all older population. This study will not be able to account for effect of intervention and change beyond the study

For peer review only

duration, making it challenging to evaluate the long-term sustainability of the intervention. By nature, it is not possible to blind participant for exercise intervention, and this may have effect on the participant lifestyle choices beyond what can be captured with the study.

Ethics and disseminations

This protocol and the informed consent forms have been reviewed and approved by the applicable ethical committees of the *Azienda Ospedaliera Universitaria Integrata* of Verona with respect to scientific content and compliance with applicable research and human subjects' regulation. Thereby, this protocol is ethically conducted following the last revision of the Declaration of Helsinki as well as the Declaration of Oviedo. The protocol of the study is designed and will be conducted in order to adhere to Good Clinical Practice principles and procedures and in compliance with the Italian legislation, as described in the following documents and accepted by the investigators of the study with their signature: a) ICH Harmonized Tripartite Guidelines for Good Clinical Practice 1996; b) Directive 91/507/EEC, The Rules Governing Medicinal Products in the European Community; c) D. L.vo n.211 of 24th June 2003; d) D. L.vo n.200 6th November 2007; e) D.M. 21st December 2007. NIH Clinical trial identification ID and number: JPI- ERAHDL NCT05343611.

Consent, confidentiality, declaration of interests

A member of the research team will introduce the trial to individuals who show an interest in taking part in the study and their caregivers. Individuals will also receive information sheets. A member of the research team will discuss the trial with potential participants and their caregivers considering the information provided in the information sheets. Individuals and caregivers will then be able to have an informed discussion with the member of the research team who will obtain written informed consent from the persons willing to participate in the trial. All study-related information will be stored securely at the study site. All participant information will be stored in locked file cabinets in area with limited access. All laboratory specimens, reports, data collection, process and administrative forms will be identified by a coded ID number only to maintain participant confidentiality. All records that contain names or other personnel identifier will be stored separately from study records identified by code number. All local databases will be secured with password-protected access systems.

For peer review only

Participants information will be stored for 10 years. Forms, lists, logbook, appointment books, and any other listing that link to participant ID numbers to other identifying information will be stored in a separate, locked file in an area with limited access.

Conflict of Interest

None declared.

Data access

Access to the database to start a data entry session is protected by a username and password. The following precautionary measures are taken to ensure data privacy and to prevent data manipulation and loss: i) Access to data is restricted to authorized members only. The authorized members are the researchers directly involved in the study who will collect, analyze, and interpret the data; ii) The network is protected by a firewall; iii) Internet connection is encrypted with a digital certificate (SSL technology); iv) The database is on a server, password protected, which is changed periodically; v) Access to the database is password protected and is accessible only to those responsible for the center; vi) Periodic backups are performed. vii) and places of conservation (e.g., The paper materials related to clinical evaluations will be stored in cabinets, whose keys will be in possession only of the persons authorized by the persons in charge of the study). Personal data of the subjects will be exclusively accessible for investigators and collaborators/co-investigators, monitors and auditors of the Promoter, and/or for the competent authority, in agreement with what is included in the Informed consent. The Coordinator and the Investigators will inform the patients in a clear and thorough way about the modalities of personal data treatment before their participation to the experiment, as established by the applicable Privacy Laws.

Dissemination policy

A consortium agreement has been established for the dissemination policy. Each Unit is encouraged to publish in written form, oral presentation or making public in any other form (including electronic publication on the internet), hereinafter referred to as the “Publication”, the results of the Project, including but not limited to solely generated Foreground, after providing a copy of the draft Publication to the other Parties at least thirty calendar days prior to submission for publication or disclosure. The other parties may, by giving written notice to the Unit requesting the Publication (a “Confidentiality Notice"), require the publishing Unit to: (a) amend the Publication in order to remove

For peer review only

any of its Background that is Confidential Information for the duration of the confidentiality obligation, or to remove any of its Foreground; or (b) amend the Publication if it can reasonably demonstrate that the manuscript compromises disproportionally its academic or legitimate interests. The other Unit shall give such Confidentiality Notice within thirty calendar days after receipt of the draft Publication. The Confidentiality Notice shall contain specific details to be amended and the Party seeking disclosure shall delete said specific details in as far as they comprise the other Units's Confidential Information from the Publication or amend said specific details as not to Consortium Agreement " Choko-Age project" disproportionally harm the requesting Unit's academic or legitimate interests. If no objection is made within the time limit stated above, the Publication is permitted.

If an objection has been raised the involved Units shall discuss how to overcome the justified grounds for the objection on a timely basis (for example by amendment to the planned Publication before publication). The objecting Unit shall not unreasonably continue the opposition if appropriate actions are performed following the discussion. In any event, any delays of a Publication shall not exceed a period of more than 90 calendar days from the date of the notification from the Unit seeking disclosure. Contributors to the results obtained through work on the Project published shall be named and accredited in the Publication in accordance with academic custom. Other aspects concerning result dissemination and the communications strategy of the project are presented in the “Communication and Dissemination plan” enclosed as Annex IV to the present Agreement.

Author contributions

AP and MI where the co-lead and written the protocol according to the project descriptive, created figures and structured the manuscripts. FG as the PI of the project have contributed equally. All other authors of Choko-Age collaborators all contributed to the conception and design of the work, data acquisition, drafting and revising the study and are accountable for its intellectual content. This is the first version of the protocol submitted to BMJ Open.

Choko-Age Collaborators: Anna Pedrinolla, Masoud Isanejad, Cinzia Antognelli, Desirée Bartolini, Consuelo Borras, Valentina Cavedon, Gabriele Di Sante, Anna Migni, Cristina Mas-Bargues, Chiara Milanese, Claudia Baschirotto, Roberto Modena,

For peer review only

Alessandra Pistilli, Mario Rende, Federico Schena , Anna Maria Stabile, Nicola Vincenzo Telesa , Sara Tortorella, Kay Hemmings, Jose Vina , Eivind Wang, Anne McArdle, Malcolm J Jackson, Massimo Venturelli, and Francesco Galli.

Funding statement

To intervene with high-calory micronutrient-rich diet in elderly patients at risk of PEM could be problematic for different reasons including the difficulty to find palatable food items suitable to satisfy these nutritional criteria. The need for specific innovation in this area of food industry and human nutrition has recently been addressed in the call PREVNUT promoted by the "Joined Programming Initiatives-Healthy Diet for a Healthy Life" (JPI-HDHL) supported by the EU program Horizon 2020 under the umbrella of ERA-Net/ERA-HDHL and the international network of funding organizations reported in (https://www.healthydietforhealthylife.eu/index.php/call-activities/calls/98-calls-site-restyling/583-era-hdhl-2020). This project has received funding from MUR, Ministero dell'Università e della Ricerca (DD17080-24/11/2021) (Italy); Instituto de Salud Carlos III (ISCIII) (Ref. AC20/00026) (Spain); RCN, The Research Council of Norway (Norway) and UKRI, Biotechnology and Biological Sciences Research Council (BBSRC) (grant number BB/V019821/1) (UK), under the umbrella of the European Joint Programming Initiative "A Healthy Diet for a Healthy Life" (JPI HDHL) and of the ERA-NET Cofund ERA-HDHL (GA N° 696295 of the EU Horizon 2020 Research and Innovation Programme).

References

1. Leij-Halfwerk S, Verwijs MH, van Houdt S, Borkent JW, Guaitoli PR, Pelgrim T, et al. Prevalence of protein-energy malnutrition risk in European older adults in community, residential and hospital settings, according to 22 malnutrition screening tools validated for use in adults ≥65 years: A systematic review and meta-analysis. Vol. 126, Maturitas. Elsevier Ireland Ltd; 2019. p. 80–9.

2. Eglseer D, Eminovic S, Lohrmann C. Association between sarcopenia and nutritional status in older adults: A systematic literature review. Vol. 42, Journal of Gerontological Nursing. Slack Incorporated; 2016. p. 33–41.

3. Mathewson SL, Azevedo PS, Gordon AL, Phillips BE, Greig CA. Overcoming protein-energy malnutrition in older adults in the residential care setting: A narrative review of causes and interventions. Ageing Res Rev [Internet]. 2021;70(May):101401. Available from: https://doi.org/10.1016/j.arr.2021.101401

4. Anderson R, Weindruch R. Metabolic reprogramming in dietary restriction. Vol. 35, Interdisciplinary Topics in Gerontology. Interdiscip Top Gerontol; 2007. p. 18–38.

5. Droogsma E, van Asselt D, de Deyn PP. Gewichtsverlust und Unterernährung bei selbstständig lebenden Patienten mit Alzheimer-Demenz: Von populationsbasierten Studien zum klinischen Management. Z Gerontol Geriatr. 2015;48(4):318–24.

6. Martocchia A, Gallucci M, Noale M, Maggi S, Cassol M, Stefanelli M, et al. The increased cortisol levels with preserved rhythmicity in aging and its relationship with dementia and metabolic syndrome. Aging Clin Exp Res. 2022;

7. Venturelli M, Cè E, Limonta E, Muti E, Scarsini R, Brasioli A, et al. Possible Predictors of Involuntary Weight Loss in Patients with Alzheimer's Disease. PLoS One. 2016;

8. Ouanes S, Popp J. High cortisol and the risk of dementia and alzheimer's disease: A review of the literature [Internet]. Vol. 11, Frontiers in Aging Neuroscience. Frontiers Media S.A.; 2019 [cited 2022 Jun 15]. Available from: https://pubmed.ncbi.nlm.nih.gov/30881301/

For peer review only

9. Oudbier SJ, Goh J, Looijaard SMLM, Reijnierse EM, Meskers CGM, Maier AB. Pathophysiological Mechanisms Explaining the Association Between Low Skeletal Muscle Mass and Cognitive Function. The Journals of Gerontology: Series A [Internet]. 2022 Oct 6 [cited 2022 Nov 5];77(10). Available from: https://pubmed.ncbi.nlm.nih.gov/35661882/

10. Beura SK, Dhapola R, Panigrahi AR, Yadav P, Reddy DH, Singh SK. Redefining oxidative stress in Alzheimer's disease: Targeting platelet reactive oxygen species for novel therapeutic options. Vol. 306, Life Sciences. Elsevier Inc.; 2022.

11. Strasser B, Wolters M, Weyh C, Krüger K, Ticinesi A. The effects of lifestyle and diet on gut microbiota composition, inflammation and muscle performance in our aging society. Nutrients. 2021;13(6).

12. Chung E, Mo H, Wang S, Zu Y, Elfakhani M, Rios SR, et al. Potential roles of vitamin E in age-related changes in skeletal muscle health. Nutrition Research. 2018;49:23–36.

13. Khor SC, Abdul Karim N, Wan Ngah WZ, Mohd Yusof YA, Makpol S. Vitamin e in sarcopenia: Current evidences on its role in prevention and treatment. Oxid Med Cell Longev. 2014;2014.

14. Nikawa T, Ulla A, Sakakibara I. Polyphenols and their effects on muscle atrophy and muscle health. Molecules. 2021;26(16).

15. Thoma A, Akter-Miah T, Reade RL, Lightfoot AP. Targeting reactive oxygen species (ROS) to combat the age-related loss of muscle mass and function. Biogerontology. 2020;21(4):475–84.

16. Wang E, Nyberg SK, Hoff J, Zhao J, Leivseth G, Tørhaug T, et al. Impact of maximal strength training on work efficiency and muscle fiber type in the elderly: Implications for physical function and fall prevention. Exp Gerontol [Internet]. 2017;91:64–71. Available from: http://dx.doi.org/10.1016/j.exger.2017.02.071

17. Pedrinolla A, Schena F, Venturelli M. Resilience to Alzheimer ' s Disease : The Role of Physical Activity. 2017;546–53.

For peer review only

18. Torvild Kittilsen H, Ingegerdsson Freberg B, Nicolaisen I, Maria Støa E, Bratland-Sanda S, Helgerud J, et al. Responses to Maximal Strength Training in Different Age and Gender Groups. Front Physiol [Internet]. 2021;12:636972. Available from: www.frontiersin.org

19. StØren Ø, Helgerud J, SÆbØ M, StØa EM, Bratland-Sanda S, Unhjem RJ, et al. The Effect of Age on the V-O2max Response to High-Intensity Interval Training. Med Sci Sports Exerc. 2017 Jan 1;49(1):78–85.

20. Helgerud J, Wang E, Mats ·, Mosti P, Øystein ·, Wiggen N, et al. Plantar Xexion training primes peripheral arterial disease patients for improvements in cardiac function. Eur J Appl Physiol. 2009;106:207–15.

21. Mosti MP, Wang E, Wiggen ØN, Helgerud J, Hoff J. Concurrent strength and endurance training improves physical capacity in patients with peripheral arterial disease. Scand J Med Sci Sports [Internet]. 2011;21:e308–14. Available from: https://onlinelibrary.wiley.com/doi/10.1111/j.1600-0838.2011.01294.x

22. Liu S, Meng F, Zhang D, Shi D, Zhou J, Guo S, et al. Lonicera caerulea Berry Polyphenols Extract Alleviates Exercise Fatigue in Mice by Reducing Oxidative Stress, Inflammation, Skeletal Muscle Cell Apoptosis, and by Increasing Cell Proliferation. Front Nutr. 2022;9:853225.

23. Mock JT, Chaudhari K, Sidhu A, Sumien N. The influence of vitamins E and C and exercise on brain aging. Exp Gerontol. 2017;94:69–72.

24. Galli F, Azzi A, Birringer M, Cook-Mills JM, Eggersdorfer M, Frank J, et al. Vitamin E: Emerging aspects and new directions. Vol. 102, Free Radical Biology and Medicine. Elsevier Inc.; 2017. p. 16–36.

25. Ammar A, Mounaturki, Trabelsi K, Bragazzi NL, Boukhris O, Bouaziz M, et al. Effects of natural polyphenol-rich pomegranate juice on the acute and delayed response of Homocysteine and steroidal hormones following weightlifting exercises: A double-blind, placebo-controlled trial. J Int Soc Sports Nutr. 2020 Mar 6;17(1).

For peer review only

26. Brobakken MF, Nyg ard M, Morken G, Reitan SK, Heggelund J, Wang E, et al. Aerobic interval training in standard treatment of out-patients with schizophrenia: a randomized controlled trial. Acta Psychiatr Scand [Internet]. 2019;140:498–507. Available from: https://onlinelibrary.wiley.com/doi/10.1111/acps.13105

27. Tøien T, Unhjem R, Øren TS, Kvellestad ACG, Hoff J, Wang E. Neural Plasticity with Age: Unilateral Maximal Strength Training Augments Efferent Neural Drive to the Contralateral Limb in Older Adults. Journals of Gerontology - Series A Biological Sciences and Medical Sciences. 2018;73(5):596–602.

28. Hart NH, Nimphius S, Spiteri T, Cochrane JL, Newton RU. Segmental musculoskeletal examinations using dual-energy X-ray absorptiometry (DXA): Positioning and analysis considerations. J Sports Sci Med. 2015 Sep 1;14(3):620–6.

29. Franchi M V., Fitze DP, Hanimann J, Sarto F, Spörri J. Panoramic ultrasound vs. MRI for the assessment of hamstrings cross-sectional area and volume in a large athletic cohort. Sci Rep. 2020 Dec 1;10(1).

30. Trinity JD, Kwon OS, Broxterman RM, Gifford JR, Kithas AC, Hydren JR, et al. The role of the endothelium in the hyperemic response to passive leg movement: looking beyond nitric oxide. American Journal of Physiology-Heart and Circulatory Physiology [Internet]. 2020 Dec 11 [cited 2021 Jan 5];ajpheart.00784.2020. Available from: https://journals.physiology.org/doi/10.1152/ajpheart.00784.2020

31. Venturelli M, Amann M, Layec G, Mcdaniel J, Trinity JD, Fjeldstad AS, et al. Passive leg movement-induced hyperaemia with a spinal cord lesion: Evidence of preserved vascular function. Acta Physiologica. 2014 Feb;210(2):429–39.

32. Pedrinolla A, Tamburin S, Brasioli A, Sollima A, Fonte C, Muti E, et al. An Indoor Therapeutic Garden for Behavioral Symptoms in Alzheimer's Disease: A Randomized Controlled Trial. Journal of Alzheimer's Disease. 2019;71(3).

33. Bartolini D, Marinelli R, Giusepponi D, Galarini R, Barola C, Stabile AM, et al. Alpha-tocopherol metabolites (the vitamin E metabolome) and their

For peer review only

interindividual variability during supplementation. Antioxidants. 2021 Feb 1;10(2):1–16.

34. Pugh JN, Stretton C, McDonagh B, Brownridge P, McArdle A, Jackson MJ, et al. Exercise stress leads to an acute loss of mitochondrial proteins and disruption of redox control in skeletal muscle of older subjects: An underlying decrease in resilience with aging? Free Radic Biol Med. 2021 Dec 1;177:88–99.

35. Claflin DR, Larkin LM, Cederna PS, Horowitz JF, Alexander NB, Cole NM, et al. Effects of high-and low-velocity resistance training on the contractile properties of skeletal muscle fibers from young and older humans. J Appl Physiol [Internet]. 2011;111:1021–30. Available from: http://www.jap.org

36. Vikberg S, Sörlén N, Brandén L, Johansson J, Nordström A, Hult A, et al. Effects of Resistance Training on Functional Strength and Muscle Mass in 70-Year-Old Individuals With Pre-sarcopenia: A Randomized Controlled Trial. J Am Med Dir Assoc. 2019;20(1):28–34.

37. Distefano G, Goodpaster BH. Effects of exercise and aging on skeletal muscle. Cold Spring Harb Perspect Med. 2018;8(3).

For peer review only

Figures legends

Figure 1. Study design and Individual's involvement. Flow chart of the study design showing information on enrolment, group assignment, follow-up, and data analysis (panel A) together with the flow chart of individuals involvement (panel B).

Figure 2. Methods of primary, secondary, and explorative outcomes. Primary outcomes include: body composition, specifically free-fat mass in the lower limbs, measured by means of DXA. Another primary outcome consists of maximal voluntary activation measured by MVS+IT+RT technique. Secondary outcomes include cross sectional area of the quadriceps muscle and pinnation angle, salivary cortisol, measured by means of chemiluminescence, and biological outcomes such as: thiols, mRNA and proteomics, vitamin-E, untargeted lipidomic, glycoxidation, hormones, cytokines, epicatechin, lipid peroxidation and protein oxidation. Furthermore, secondary outcomes include mitochondrial respiration measured on muscle fibbers, single fibre force, fibber typing, cytokines, transcriptomics, and proteomic measured in the muscle fibbers. Exploratory outcomes include vitamin-e measured in muscle fibber, and faecal microbiome.

For peer review only

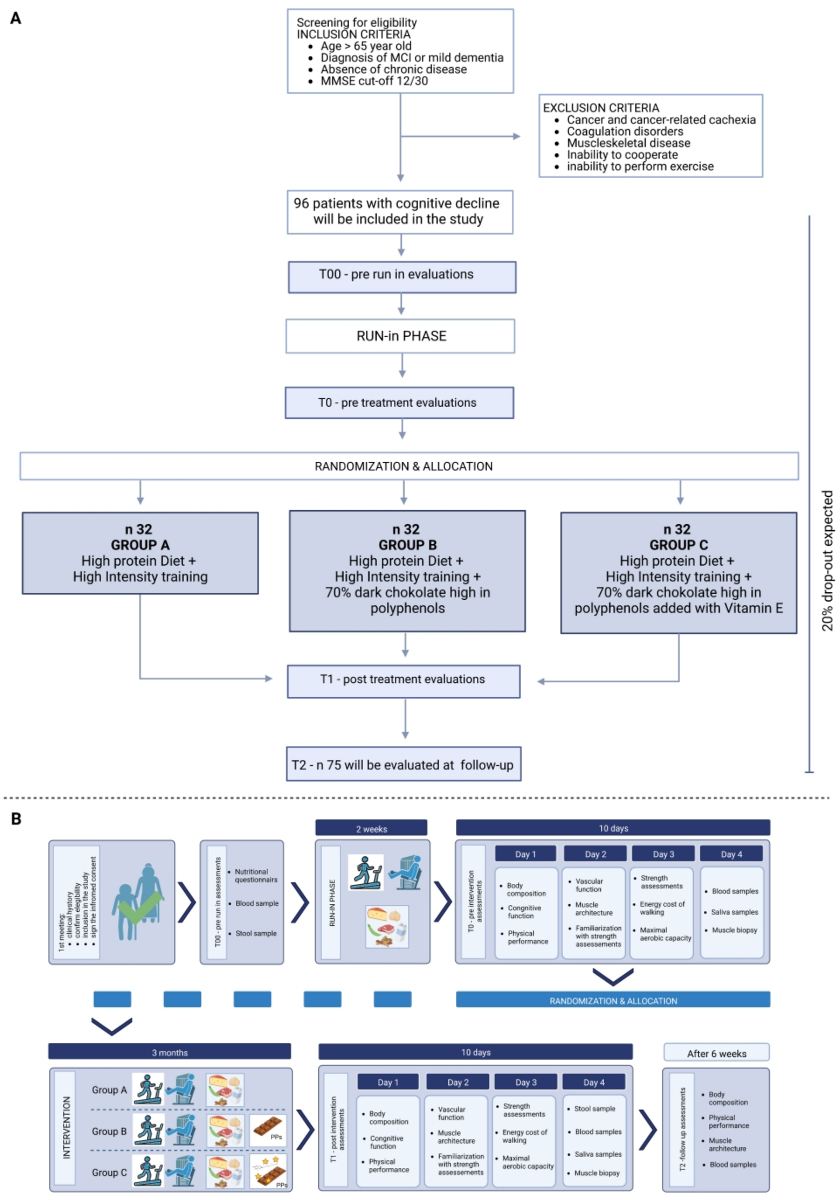

Individual timeline

305x425mm (300 x 300 DPI)

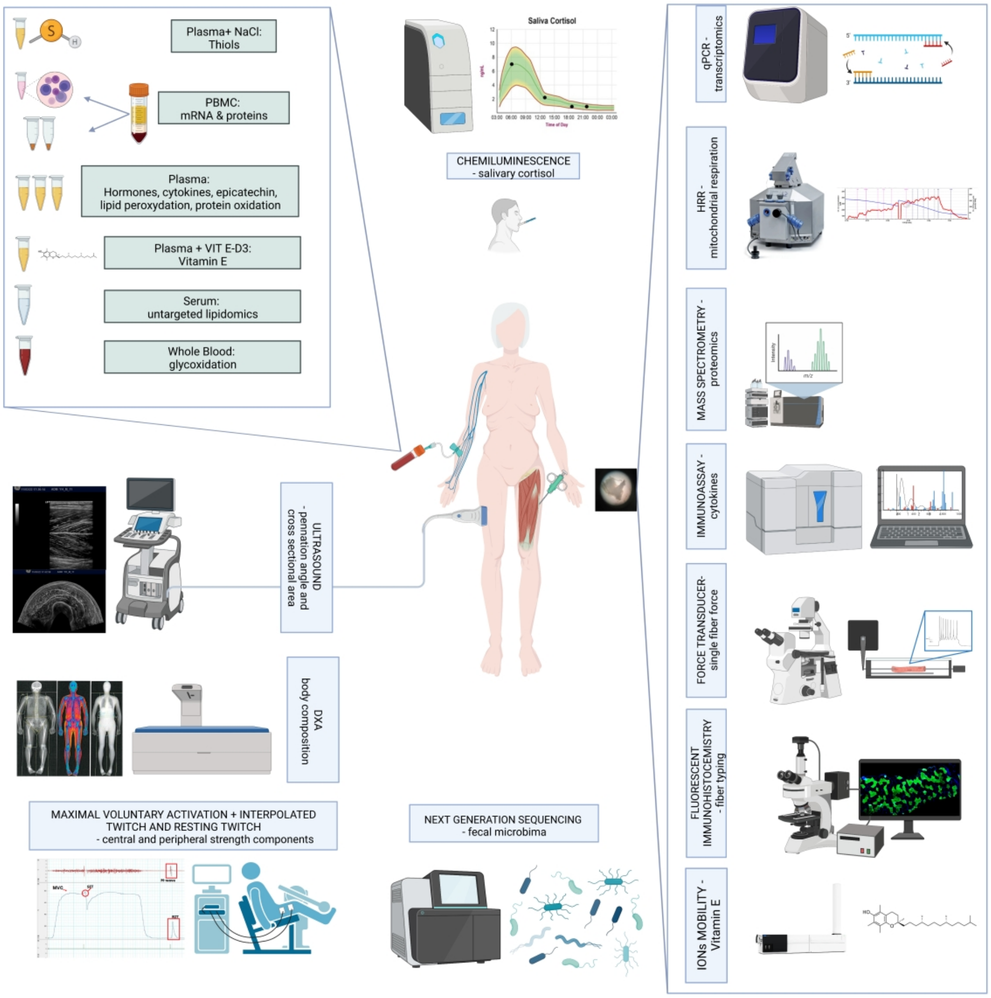

Study graphical abstract for clinical and laboratory endpoints

406x406mm (300 x 300 DPI)

Reporting checklist for protocol of a clinical trial.

Based on the SPIRIT guidelines.

Instructions to authors

Complete this checklist by entering the page numbers from your manuscript where readers will find each of the items listed below.

Your article may not currently address all the items on the checklist. Please modify your text to include the missing information. If you are certain that an item does not apply, please write "n/a" and provide a short explanation.

Upload your completed checklist as an extra file when you submit to a journal.

In your methods section, say that you used the SPIRITreporting guidelines, and cite them as:

Chan A-W, Tetzlaff JM, Gøtzsche PC, Altman DG, Mann H, Berlin J, Dickersin K, Hróbjartsson A, Schulz KF, Parulekar WR, Krleža-Jerić K, Laupacis A, Moher D. SPIRIT 2013 Explanation and Elaboration: Guidance for protocols of clinical trials. BMJ. 2013;346:e7586

		Reporting Item	Page Number
**Administrative information**			
Title	#1	Descriptive title identifying the study design, population, interventions, and, if applicable, trial acronym	1
Trial registration	#2a	Trial identifier and registry name. If not yet registered, name of intended registry	4
Trial registration: data set	#2b	All items from the World Health Organization Trial Registration Data Set	9
Protocol version	#3	Date and version identifier	11
Funding	#4	Sources and types of financial, material, and other support	11
Roles and responsibilities: contributorship	#5a	Names, affiliations, and roles of protocol contributors	10

For peer review only

Roles and responsibilities: sponsor contact information	#5b	Name and contact information for the trial sponsor	NA
Roles and responsibilities: sponsor and funder	#5c	Role of study sponsor and funders, if any, in study design; collection, management, analysis, and interpretation of data; writing of the report; and the decision to submit the report for publication, including whether they will have ultimate authority over any of these activities	11
Roles and responsibilities: committees	#5d	Composition, roles, and responsibilities of the coordinating centre, steering committee, endpoint adjudication committee, data management team, and other individuals or groups overseeing the trial, if applicable (see Item 21a for data monitoring committee)	9
**Introduction**			
Background and rationale	#6a	Description of research question and justification for undertaking the trial, including summary of relevant studies (published and unpublished) examining benefits and harms for each intervention	1
Background and rationale: choice of comparators	#6b	Explanation for choice of comparators	1
Objectives	#7	Specific objectives or hypotheses	2
Trial design	#8	Description of trial design including type of trial (eg, parallel group, crossover, factorial, single group), allocation ratio, and framework (eg, superiority, equivalence, non-inferiority, exploratory)	3
**Methods: Participants, interventions, and outcomes**			
Study setting	#9	Description of study settings (eg, community clinic, academic hospital) and list of countries where data will be collected. Reference to where list of study sites can be	3

For peer review only

		obtained	
Eligibility criteria	#10	Inclusion and exclusion criteria for participants. If applicable, eligibility criteria for study centres and individuals who will perform the interventions (eg, surgeons, psychotherapists)	4
Interventions: description	#11a	Interventions for each group with sufficient detail to allow replication, including how and when they will be administered	4
Interventions: modifications	#11b	Criteria for discontinuing or modifying allocated interventions for a given trial participant (eg, drug dose change in response to harms, participant request, or improving / worsening disease)	4
Interventions: adherance	#11c	Strategies to improve adherence to intervention protocols, and any procedures for monitoring adherence (eg, drug tablet return; laboratory tests)	5
Interventions: concomitant care	#11d	Relevant concomitant care and interventions that are permitted or prohibited during the trial	4
Outcomes	#12	Primary, secondary, and other outcomes, including the specific measurement variable (eg, systolic blood pressure), analysis metric (eg, change from baseline, final value, time to event), method of aggregation (eg, median, proportion), and time point for each outcome. Explanation of the clinical relevance of chosen efficacy and harm outcomes is strongly recommended	5.6
Participant timeline	#13	Time schedule of enrolment, interventions (including any run-ins and washouts), assessments, and visits for participants. A schematic diagram is highly recommended (see Figure)	5
Sample size	#14	Estimated number of participants needed to achieve study objectives and how it was determined, including clinical and statistical assumptions supporting any sample size calculations	8
Recruitment	#15	Strategies for achieving adequate participant enrolment to reach target sample size	8

For peer review only

**Methods: Assignment of interventions (for controlled trials)**			
Allocation: sequence generation	#16a	Method of generating the allocation sequence (eg, computer-generated random numbers), and list of any factors for stratification. To reduce predictability of a random sequence, details of any planned restriction (eg, blocking) should be provided in a separate document that is unavailable to those who enrol participants or assign interventions	8
Allocation concealment mechanism	#16b	Mechanism of implementing the allocation sequence (eg, central telephone; sequentially numbered, opaque, sealed envelopes), describing any steps to conceal the sequence until interventions are assigned	8
Allocation: implementation	#16c	Who will generate the allocation sequence, who will enrol participants, and who will assign participants to interventions	8
Blinding (masking)	#17a	Who will be blinded after assignment to interventions (eg, trial participants, care providers, outcome assessors, data analysts), and how	8
Blinding (masking): emergency unblinding	#17b	If blinded, circumstances under which unblinding is permissible, and procedure for revealing a participant's allocated intervention during the trial	8
**Methods: Data collection, management, and analysis**			
Data collection plan	#18a	Plans for assessment and collection of outcome, baseline, and other trial data, including any related processes to promote data quality (eg, duplicate measurements, training of assessors) and a description of study instruments (eg, questionnaires, laboratory tests) along with their reliability and validity, if known. Reference to where data collection forms can be found, if not in the	9

For peer review only

		protocol	
Data collection plan: retention	#18b	Plans to promote participant retention and complete follow-up, including list of any outcome data to be collected for participants who discontinue or deviate from intervention protocols	9
Data management	#19	Plans for data entry, coding, security, and storage, including any related processes to promote data quality (eg, double data entry; range checks for data values). Reference to where details of data management procedures can be found, if not in the protocol	9
Statistics: outcomes	#20a	Statistical methods for analysing primary and secondary outcomes. Reference to where other details of the statistical analysis plan can be found, if not in the protocol	5
Statistics: additional analyses	#20b	Methods for any additional analyses (eg, subgroup and adjusted analyses)	8
Statistics: analysis population and missing data	#20c	Definition of analysis population relating to protocol non-adherence (eg, as randomised analysis), and any statistical methods to handle missing data (eg, multiple imputation)	8
**Methods: Monitoring**			
Data monitoring: formal committee	#21a	Composition of data monitoring committee (DMC); summary of its role and reporting structure; statement of whether it is independent from the sponsor and competing interests; and reference to where further details about its charter can be found, if not in the protocol. Alternatively, an explanation of why a DMC is not needed	8
Data monitoring: interim analysis	#21b	Description of any interim analyses and stopping guidelines, including who will have access to these interim results and make the final decision to terminate the trial	8
Harms	#22	Plans for collecting, assessing, reporting, and managing solicited and spontaneously reported adverse events and other unintended effects of trial interventions or trial conduct	9
Auditing	#23	Frequency and procedures for auditing trial conduct, if	9

For peer review only

		any, and whether the process will be independent from investigators and the sponsor	
**Ethics and dissemination**			
Research ethics approval	#24	Plans for seeking research ethics committee / institutional review board (REC / IRB) approval	9
Protocol amendments	#25	Plans for communicating important protocol modifications (eg, changes to eligibility criteria, outcomes, analyses) to relevant parties (eg, investigators, REC / IRBs, trial participants, trial registries, journals, regulators)	11
Consent or assent	#26a	Who will obtain informed consent or assent from potential trial participants or authorised surrogates, and how (see Item 32)	9
Consent or assent: ancillary studies	#26b	Additional consent provisions for collection and use of participant data and biological specimens in ancillary studies, if applicable	9
Confidentiality	#27	How personal information about potential and enrolled participants will be collected, shared, and maintained in order to protect confidentiality before, during, and after the trial	9
Declaration of interests	#28	Financial and other competing interests for principal investigators for the overall trial and each study site	9
Data access	#29	Statement of who will have access to the final trial dataset, and disclosure of contractual agreements that limit such access for investigators	9
Ancillary and post trial care	#30	Provisions, if any, for ancillary and post-trial care, and for compensation to those who suffer harm from trial participation	NA
Dissemination policy: trial results	#31a	Plans for investigators and sponsor to communicate trial results to participants, healthcare professionals, the public, and other relevant groups (eg, via publication, reporting in results databases, or other data sharing arrangements), including any publication restrictions	10
Dissemination policy:	#31b	Authorship eligibility guidelines and any intended use of	10

For peer review only

authorship		professional writers	
Dissemination policy: reproducible research	#31c	Plans, if any, for granting public access to the full protocol, participant-level dataset, and statistical code	10

Appendices

Informed consent materials	#32	Model consent form and other related documentation given to participants and authorised surrogates	NA
Biological specimens	#33	Plans for collection, laboratory evaluation, and storage of biological specimens for genetic or molecular analysis in the current trial and for future use in ancillary studies, if applicable	NA

The SPIRIT Explanation and Elaboration paper is distributed under the terms of the Creative Commons Attribution License CC-BY-NC. This checklist was completed on 26. January 2023 using https://www.goodreports.org/, a tool made by the EQUATOR Network in collaboration with Penelope.ai

For peer review only
